# Examining Caregiver Practices During Adolescent Outpatient Alcohol Use and Co-Occurring Mental Health Treatment: Protocol for a Dyadic Ecological Momentary Assessment Study

**DOI:** 10.2196/63399

**Published:** 2024-12-20

**Authors:** Samuel N Meisel, Aaron Hogue, John F Kelly, Elizabeth McQuaid, Robert Miranda Jr

**Affiliations:** 1 Department of Psychological and Brain Sciences Boston University Boston, MA United States; 2 Partnership to End Addiction New York, NY United States; 3 Recovery Research Institute, Center for Addiction Medicine, Massachusetts General Hospital & Harvard Medical School Boston, MA United States; 4 Department of Psychiatry and Human Behavior, Warren Alpert Medical School of Brown University Providence, RI United States; 5 Center for Alcohol and Addiction Studies Department of Psychiatry and Human Behavior, Warren Alpert Medical School of Brown University, Brown University Providence, RI United States; 6 E. P. Bradley Hospital Riverside, RI United States

**Keywords:** adolescent, caregiver, ecological momentary assessment, alcohol, co-occurring disorders treatment, treatment, older adult, aging, alcohol use, mental health, assessment, protocol, alcohol use disorder, drinking, substance use, data collection

## Abstract

**Background:**

Caregiver-involved treatments for adolescents with alcohol use disorder and co-occurring disorders (AUD+CODs) are associated with the best treatment outcomes. Understanding what caregiving practices during treatment improve core adolescent treatment targets may facilitate the refinement and scalability of caregiver-involved interventions. Caregiving is dynamic, varying by context, affect, and adolescent behavior. Caregiver-involved treatments seek to change momentary interactions between caregivers and their adolescents. Accordingly, this protocol outlines a dyadic ecological momentary assessment (EMA) study to examine caregiving practices during AUD+CODs treatment and their associations with adolescent core treatment targets (eg, alcohol craving and use, motivation to reduce or stop drinking, and internalizing and externalizing symptoms).

**Objective:**

This paper aims to describe the methods for examining momentary caregiving practices and adolescent core treatment targets during adolescent outpatient AUD+CODs treatment.

**Methods:**

We will recruit 75 caregiver-adolescent dyads from outpatient mental health clinics providing AUD+CODs treatment. Eligible families will have an adolescent who (1) is aged between 13 and 18 years; (2) meets the *Diagnostic and Statistical Manual of Mental Disorders, Fifth Edition*, diagnostic criteria for AUD; (3) is enrolled in outpatient treatment at the time of recruitment; and (4) has a legal guardian willing to participate in the study. Caregivers and adolescents will complete an eligibility screening, followed by a baseline assessment during or as close as possible to the second week of treatment. During the baseline assessment, caregivers and adolescents will receive formal training in EMA procedures. Next, caregivers and adolescents will complete a 15-week EMA burst design consisting of three 21-day EMA periods with 3-week breaks between periods. Throughout the study, participants will also complete weekly reports regarding the skills learned or practiced during therapy. The three overarching aims to the proposed study are as follows: (1) examine momentary caregiving practices (eg, support, monitoring, substance use communication quality) and their associations with core treatment targets, (2) examine how these associations change throughout treatment, and (3) examine whether a caregiver report of learning or practicing parenting- or family-focused behaviors in treatment sessions is associated with changes in the use of caregiving practices in daily life.

**Results:**

The proposed study was informed by a pilot study assessing the feasibility and acceptability of dyadic EMA during adolescent AUD+COD treatment. Some benchmarks were met during this study (eg, ≥80% caregiver retention rate), although most benchmarks were not (eg, adolescent [772/1622, 47.6%] and caregiver [1331/1881, 70.76%] random prompt compliance was below the ≥80% target). Data collection is anticipated to begin in December of 2024. The proposed study is designed to be completed over 3 years.

**Conclusions:**

Examining momentary caregiving practices using EMA has important implications for refining and scaling caregiver-involved interventions for AUD+CODs so that families who would benefit from caregiver-involved treatments can have access to them.

**International Registered Report Identifier (IRRID):**

PRR1-10.2196/63399

## Introduction

### Adolescent Alcohol Use Disorder and Co-Occurring Disorders

Caregivers are the ultimate role models for children. Every word, movement, and action has an effect. No other person or outside force has a greater influence on a child than the caregiver.

Bob Keeshan

More than 750,000 adolescents in the United States aged between 12 and 17 years have alcohol use disorder (AUD), according to current estimates [1]. Among adolescents who present for AUD treatment, >80% have a co-occurring mental health diagnosis [2,3]. Youth with AUD and co-occurring disorders (AUD+CODs) exhibit poor family functioning, low treatment motivation and engagement, poor treatment outcomes, and high rates of returning to substance use [4-6]. Although treatments for adolescents with AUD+CODs yield small, short-term benefits, caregiver-involved treatments are associated with the best outcomes [7,8]. This mirrors findings for caregiver-involved treatments for myriad adolescent mental health conditions (eg, depression and conduct disorder) [9]. Throughout this paper, we use the term *caregiver* to reflect that figures other than biological parents (eg, grandparents and stepparents) can serve as caregivers for youth in treatment. To date, limited work has examined caregiver behaviors during adolescent AUD+CODs treatment [10]. Building on theoretical and empirical research from clinical and developmental science emphasizing the importance of studying caregiving practices in the moment [11-13], this protocol describes the rationale and methods for a study seeking to address a central yet unanswered question: What specific caregiving behaviors during caregiver-involved treatment for AUD+CODs contribute to improved outcomes?

### Caregiver Involvement in AUD+CODs Treatment

Caregiver involvement in adolescent services is positively associated with adolescent treatment engagement, motivation, retention, and outcomes [9,14-20]. Moreover, adolescent AUD treatments that involve caregivers have been found to reduce co-occurring externalizing and internalizing symptoms [21-24]. These findings align with evidence that caregiving practices mediate adolescent treatment for internalizing (eg, depression and anxiety) and externalizing (eg, attention-deficit/hyperactivity disorder and oppositional defiant disorder) conditions that often co-occur with adolescent AUD [25]. Consequently, current guidelines identify caregiver-involved adolescent AUD+CODs treatment as a best practice [8].

Research examining what caregivers are doing in their routine environments during the course of treatment to promote improved youth outcomes is at a nascent stage [26,27]. Moreover, although multiple evidence-based treatments (eg, cognitive behavioral therapy, family-based therapies, and multicomponent therapies) call for the involvement of caregivers in some capacity [26,28-31], typical community-based practices for treating adolescents with AUD+CODs do not involve caregivers [8,32-34]. Structural barriers to delivering caregiver-involved treatments in outpatient settings include the high costs of the training required for family-based treatments, session frequency and duration, and highly manualized protocols with limited flexibility [8,26,35]. These barriers may affect youth considered the most vulnerable whose families have limited knowledge, access, and resources to complete caregiver-involved treatments [26,36,37]. One way to advance treatment options for youth is to identify the specific caregiving practices (ie, change-promoting parenting behaviors) during AUD+CODs treatment that most strongly relate to positive changes in core treatment targets. By isolating these behaviors, we can develop scalable and targeted caregiving interventions to maximize those treatment ingredients that yield the highest impact while mitigating required resources [38].

Unfortunately, most of what we know about how caregiving behavior relates to adolescent AUD outcomes derives from observational studies of adolescents without AUDs and not in treatment [39]. To our knowledge, only 7 studies have assessed caregiving behaviors as putative mechanisms of adolescent substance use treatment outcomes [20,40-45], and only 1 of them assessed caregiving behavior during treatment [40]. In the study, growth in parental monitoring from baseline through 12-month follow-up predicted decreased growth in substance use from baseline to 12-month follow-up [40]. Although the study provides additional support for the importance of caregiver behavior to treatment outcomes, the study design did not prospectively examine how caregiving during treatment predicts outcomes [40]. Moreover, the study, like virtually all caregiving-AUD research [39,46], operationalized caregiving via assessments spaced months apart that aggregated caregiving behaviors across weeks or months [11,47]. Harnessing the impact of caregivers to boost adolescent AUD outcomes in the most impactful and efficient way requires methods that capture how caregiving behaviors, in real time in daily life, relate to core treatment targets during therapy.

### The Need for Moment-Level Caregiving Assessments

Dynamic system theory posits that momentary interactions between a caregiver and their child create a reciprocal interaction system (ie, relationship pattern) that impacts long-term adjustment [11,48,49]. In a treatment context, this suggests that examining how caregivers act toward their children during AUD+CODs treatment is critical to understanding long-term treatment outcomes. A growing body of work from developmental science highlights the importance of studying caregiving behaviors and their associations with the indicators of adolescent adjustment at a more refined timescale [50-53]. Consistent with dynamic systems theory, these studies have found caregiving practices to vary from moment to moment depending on context, affect, and adolescent behavior [11-13]. Importantly, emerging research indicates that associations between caregiving practices and adolescent adjustment indicators do not generalize across different timescales [54]. In other words, capturing caregiver practices and adolescent adjustment on broader time courses (eg, monthly) may not representatively encode what occurs moment to moment in daily life.

Core adolescent AUD+CODs treatment targets, such as treatment motivation [55,56], alcohol craving [57-59], substance use [59,60], and internalizing and externalizing symptoms [61-63], vary considerably within and across days. Understanding improvements prompted by caregiver-involved treatments for adolescent AUD+CODs requires momentary assessments that can capture and correlate the dynamic nature of caregiving and adolescent treatment targets. Ecological momentary assessment (EMA) provides a methodology to study caregiving behaviors and evaluate their impact on adolescent functioning in real time [64,65]. EMA has been suggested to minimize recall bias, memory heuristics, and demand characteristics that can occur in caregiving research [64,66-68]. It has the unique ability to go beyond looking at how caregiving predicts behaviors across families (between-family associations) and provides rich data on how changes in a caregiver’s behavior relate to changes in their adolescent’s behavior in real time in the family’s daily life (ie, within-family associations [64]). Despite the developmental salience of these dynamic associations, no research has examined these momentary associations between caregiving practices and adolescent behavior during AUD+CODs treatment.

### The Proposed Study

The proposed protocol aims to examine caregiving practices, in the moment, during adolescent community outpatient AUD+CODs treatment. To advance our understanding of caregiving practices that promote improvements in core treatment targets, the proposed study has 3 overarching research aims and corresponding hypotheses.

Aim 1 is to examine momentary associations between caregiving practices and core treatment targets. Hypothesis 1 is that momentary *caregiver reports* of caregiver support, caregiver monitoring, substance use communication quality, and alcohol-specific caregiving practices will be positively associated with *adolescent reports* of motivation to reduce or stop drinking and positive affect and negatively associated with alcohol craving, alcohol use, and internalizing and externalizing symptoms. Momentary *caregiver reports* of caregiver-adolescent conflict will be negatively associated with *adolescent reports* of motivation to reduce or stop drinking and positive affect and positively associated with alcohol craving, alcohol use, and internalizing and externalizing symptoms.

Aim 2 is to examine changes in the strength of the associations between caregiving practices and core treatment targets over the course of treatment, consistent with treatment perspectives arguing that skill use should be more effective across time [69]. Hypothesis 2 is that aim 1 associations for caregiver support, caregiver monitoring, substance use communication quality, and alcohol-specific caregiving practices will become stronger over the course of AUD+CODs treatment as caregivers and adolescents learn and practice skills over the course of treatment. Aim 1 associations for caregiver-adolescent conflict will become weaker as caregivers and adolescents learn and practice skills to better manage conflict in the moment.

Aim 3 is to examine whether the use of family-focused therapy techniques are associated with changes in the use of caregiving practices. Hypothesis 3 is that caregiver reports of learning or practicing family-focused therapy techniques in therapy will be prospectively positively associated with increases in caregiver support, caregiver monitoring, substance use communication quality, and alcohol-specific caregiving practices and with decreases in caregiver-adolescent conflict.

## Methods

### Target Population and Recruitment

The adolescents eligible for Project Momentary Assessment of Parenting Practices (MAPP) will be those who (1) are aged between 13 and 18 years; (2) meet the *Diagnostic and Statistical Manual of Mental Disorders, Fifth Edition*, diagnostic criteria for AUD; (3) are enrolled in outpatient treatment at the time of recruitment; and (4) have a caregiver willing to participate in the study. Considering that >80% of adolescents with an AUD presenting to treatment have a co-occurring mental health condition [2,3]—and consistent with current dimensional conceptualizations of psychopathology [70]—a formal diagnosis of a co-occurring condition will not be required for eligibility. Exclusion criteria will include (1) adolescents exhibiting acute psychosis and (2) adolescents or caregivers who are unable to read or comprehend the study forms in English or Spanish (eg, consent procedures).

Seventy-five dyads will be recruited from 5 community-based mental health clinics treating adolescents with co-occurring conditions in Massachusetts. On average, these clinics have 9 (SD 7.36, range 3-20) therapists working with adolescents with co-occurring conditions and have the capacity to see 159 (SD 123.74, range 50-350) adolescents per year. Across these mental health clinics, several treatments are offered, including the adolescent community reinforcement approach (A-CRA; all 5 clinics), trauma-focused cognitive behavioral therapy (1 clinic), eye movement desensitization and reprocessing (1 clinic), and home-based services (1 clinic). A-CRA is the preferred treatment model endorsed by the Office of Youth and Young Adult Services in the Bureau of Substance Addiction Services, Massachusetts. Caregiver involvement varies across settings and treatments, with examples of caregiver involvement being weekly family therapy sessions for home-based services and 2 formal caregiver sessions in A-CRA.

Before recruiting families, the study team will provide presentations to each partnering clinic about the study so that clinicians can inform their clients about the study and answer any questions they may have. At each clinic, recruitment will occur using flyers that will be distributed to all new adolescent clients and their caregivers. In line with recent recommendations for facilitating compliance and building rapport in EMA research with adolescents [71], the flyers will also briefly introduce the Project MAPP research team and include pictures of team members. Interested families will be able to notify study staff if they are interested in participating and would like to complete an eligibility questionnaire through a QR code on the flyer. This method of recruitment was selected so that no clinic needs to provide the study team with any protected health information.

### Ethical Considerations

#### Overview

Project MAPP has been received Boston University ethics committee approval (7636E). Consent, for adolescents who are aged 18 years and caregivers, as well as parental permission and assent, for adolescents who are aged <18 years, will be obtained from all dyads before participation in Project MAPP. Adolescents and caregivers can each earn up to US $570 (refer to the next subsection for details).

#### Compensation Structure

Adolescents and their caregivers will each be compensated US $30 for completing the baseline study visit. Adolescents and their caregivers deemed ineligible during the baseline visit will each be compensated US $15 for their time. Both adolescents and their caregivers will be compensated US $1.25 per completed EMA survey during the 9 weeks of EMA and US $1.25 per morning report during non-EMA weeks. Adolescents and their caregivers will be eligible for a US $10 bonus for maintaining a weekly EMA compliance rate of ≥75%. During the weeks when the dyads are not completing EMA, they will be eligible for US $5 bonuses if their morning report compliance rate is ≥75%. Adolescents and caregivers will be compensated US $2 for each mental health update report they complete. The total possible amount a participant can earn is US $570. Adolescents and their caregivers will receive gift cards from, for example, Amazon, Walmart, and Target, as compensation. This compensation structure is similar to that of prior studies with adolescents and their caregivers [62,72].

### Procedure and Protocol

**Figure 1:**
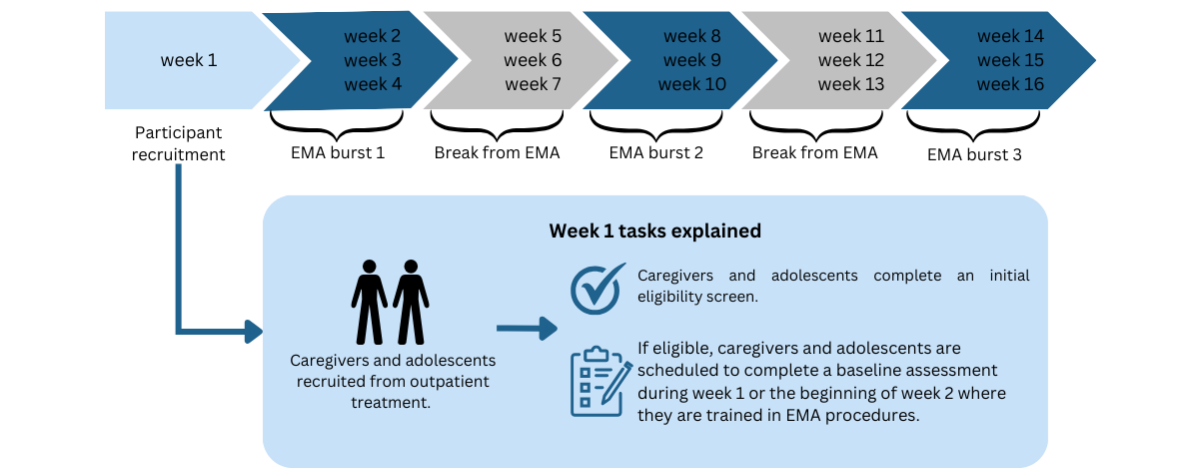
Proposed study design overview. EMA: ecological momentary assessment. *Participants will still complete morning reports and mental health update reports during the two 3-week breaks from random prompts.

**Figure 2:**
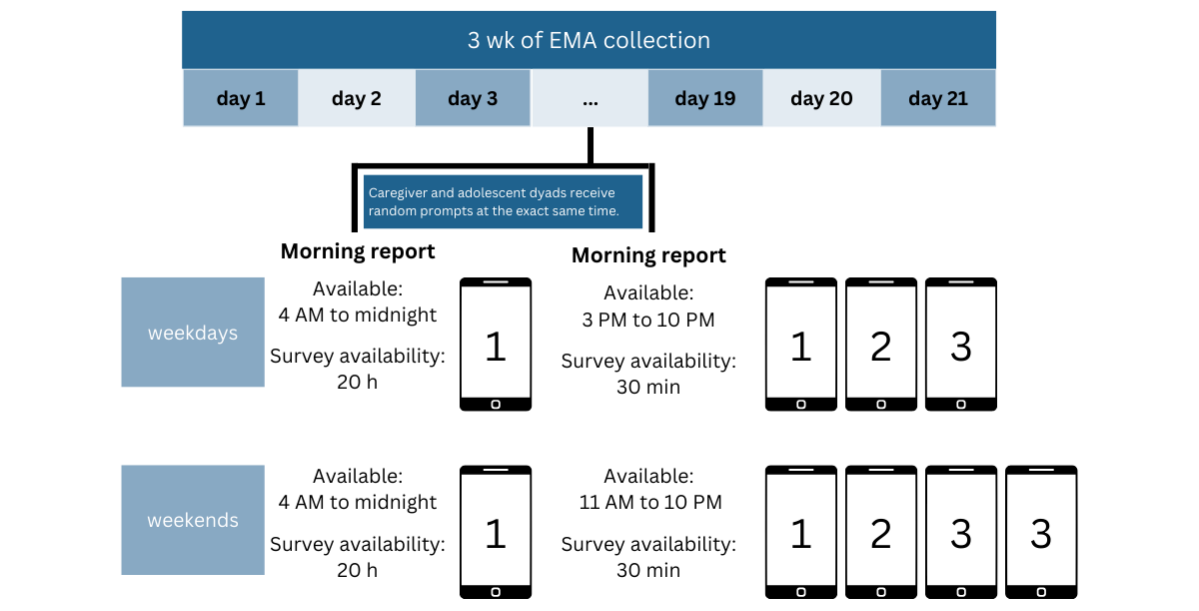
Ecological momentary assessment (EMA) procedures for the proposed study.

Figure 1 provides an overview of the study components of Project MAPP. Project MAPP consists of two components: (1) an eligibility screening and baseline assessment and (2) an EMA burst design (Figure 2). All components of Project MAPP will be conducted remotely via Health Insurance Portability and Accountability Act (HIPAA)–compliant video communication platforms.

### Baseline Study Visit

**Figure 3:**
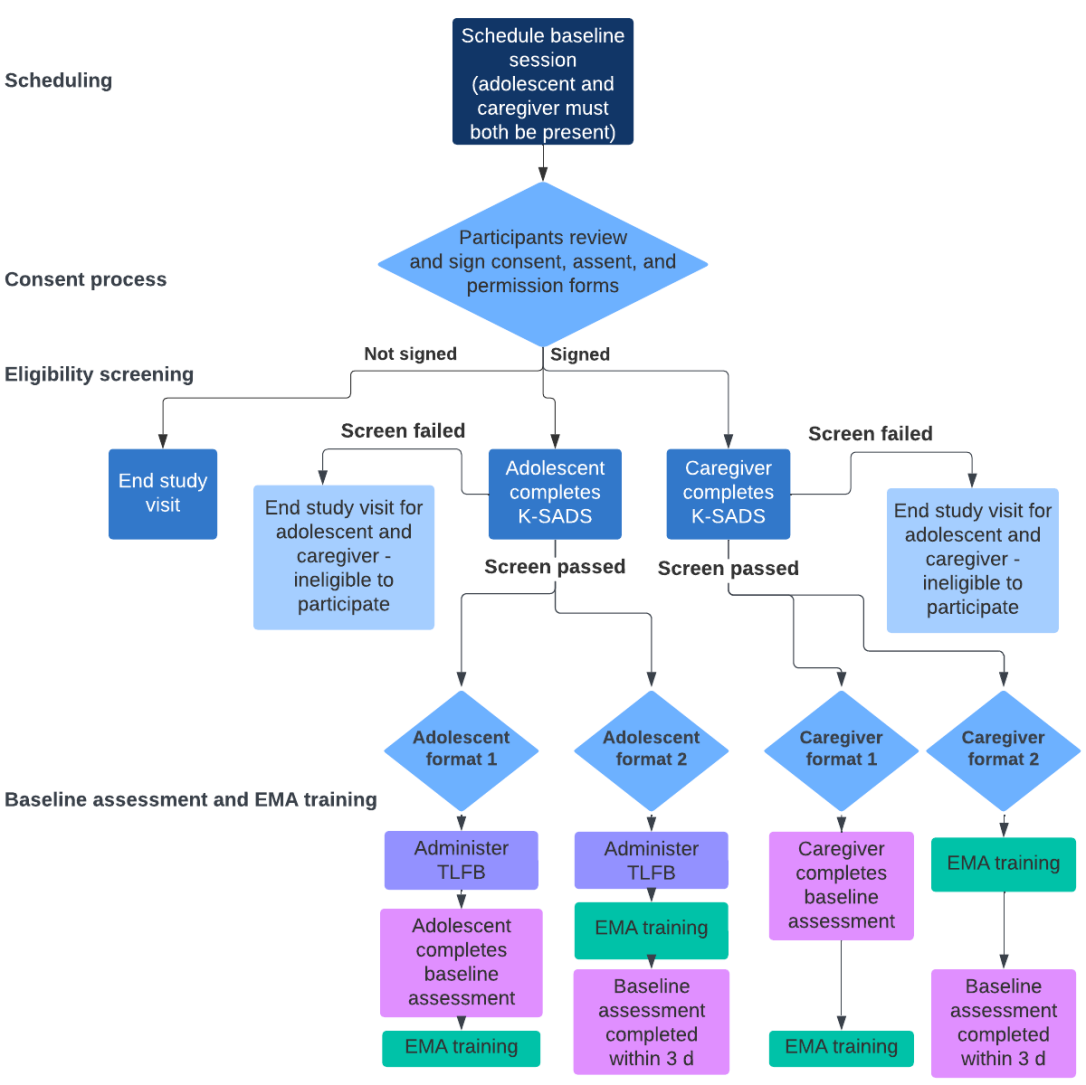
Order of procedures for the baseline study visit. EMA: ecological momentary assessment; K-SADS: Schedule for Affective Disorders and Schizophrenia for School-Aged Children; TLFB: timeline followback.

Figure 3 provides an overview of the procedures for the baseline study visit. Eligible dyads interested in participating in Project MAPP will be scheduled for their baseline assessment session during the second week of their treatment. If dyads are unable to schedule the study visits during the second week of treatment, they will be scheduled as close to the second week of treatment as possible. The baseline session will be offered in 2 formats: In format 1, dyads complete consent, assent, and parental permission forms; complete a formal assessment for AUD; complete baseline assessment measures, including a timeline followback (TLFB) interview for the adolescent; and are trained in EMA study procedures. In format 2, adolescents and caregivers complete all components of the baseline other than the baseline assessments, which they can complete at their own convenience within 72 hours of the baseline session.

These 2 options are provided to accommodate caregiver and adolescent preferences and time constraints.

For both formats, after completing the consent (for caregivers aged ≥18 y and adolescents aged 18 y), parental permission (for caregivers of adolescents aged <18 y), and assent (for adolescents aged <18 y) procedures, adolescents and caregivers will each complete the Schedule for Affective Disorders and Schizophrenia for School-Aged Children in separate web-based breakout rooms to formally assess for adolescent AUD [73]. Dyads will be notified that they are not eligible for Project MAPP if the adolescent does not meet the diagnostic criteria for AUD, and they will be compensated for their time. Eligible adolescents will complete a 30-day TLFB assessing their alcohol and other substance use (eg, cannabis and nicotine) in the past 30 days. The TLFB is a gold standard assessment for capturing alcohol and other substance use behaviors [74,75].

In format 1 of the baseline visit, caregivers and adolescents will next complete an assessment battery measuring demographic information (eg, age, gender, and family composition) as well as validated self-report measures of the EMA parenting domains and the treatment outcomes of interest (Table 1, column 3). A strength of the parenting measures selected for this study is that they contain parent- and adolescent-report versions, attenuating concerns regarding demand characteristics in parenting measures [76]. Internalizing and externalizing mental health symptoms will be measured continuously using the Child Behavior Checklist and the Youth Self-Report, which are measures from the Achenbach System of Empirically Based Assessment (Table 1) [77,78]. In format 2, adolescents and caregivers will complete these measures on their own time within 72 hours of completing the baseline visit.

**Table 1:** Baseline assessment measures and schedule of ecological momentary assessment (EMA) for the constructs of interest.

Construct			Baseline assessment measure		EMA reporter					EMA report		
					Teen		Caregiver		MR^a^			RP^b^
**Parenting**												
	Parental communication	Network of Relationships Inventory [79] McMaster Family Assessment Device [80] Parenting Styles Circumplex Inventory [81]		✓		✓					✓	
	General parenting practices	Parental Monitoring Questionnaire [82] Issues Checklist [83] and Conflict Tactics Scale [84] Alabama Parenting Questionnaire [85] Parental Locus of Control [86]		✓		✓					✓	
	Alcohol- and other substance-specific parenting practices	Substance-specific parenting measure [87,88] Alcohol-Specific Socialization Practices [89] Alcohol-Specific Communication Scale [90] Substance Use Communication Quality [91]		✓		✓		✓			✓	
**Core treatment targets**												
	Treatment motivation	University of Rhode Island Change Assessment [92]		✓		✓		✓			✓	
	Alcohol and other substance craving	Alcohol Urge Questionnaire [93] Marijuana Craving Questionnaire–Short Form [94] Questionnaire of Vaping Craving [95] Tobacco Craving Questionnaire–Short Form [96]		✓				✓			✓	
	Alcohol and other substance use	Timeline followback [97] Fagerström Test for Nicotine Dependence [98] Penn State Electronic Cigarette Dependence Index [99] Substance Problem Index–Adolescent [100]		✓		✓^c^		✓			✓	
	Internalizing and externalizing symptoms	Youth Self-Report and Child Behavior Checklist [77] Affective Reactivity Index [101]		✓		✓		✓				
**Context**												
	Location	—^d^		✓		✓					✓	
	Individuals present	—		✓		✓					✓	
	Current activity	—		✓		✓					✓	

^a^MR: morning report.

^b^RP: random prompt.

^c^Caregiver will be asked questions about their knowledge of their child’s past-day alcohol and other substance use in addition to questions about their own use.

^d^Not applicable.

For both formats 1 and 2, the last component of the baseline study visit consists of EMA training. Participants will be instructed to download the EMA software, created using a MetricWire platform (MetricWire Inc), and register for Project MAPP. Research assistants will use an EMA training manual developed for adolescent alcohol and other drug EMA research [102,103]. First, adolescents and caregivers are walked through a MetricWire demonstration survey. Research assistants walk participants through completing question types that they will encounter (eg, check boxes, typed responses, and Likert-scale items) and answer any questions that participants may have. Second, participants are led through completing a problem survey report. These surveys are intended for participants to use when they experience any difficulties with the MetricWire app. In addition, research assistants demonstrate the messaging feature of MetricWire where participants can contact any study staff if they have questions or issues with the EMA software. Third, to facilitate the reporting of standard drinks of alcohol, research assistants walk adolescents through a standard drink training on MetricWire. This survey, which is available throughout the study, includes detailed information on what constitutes a standard drink of alcohol.

As the last step of the baseline visit, research assistants will walk participants through a series of procedures intended to maximize the ease of completing the EMA protocol and EMA compliance. These procedures consist of study staff reviewing times where participants are unable to complete EMA (eg, during work or extracurricular activities) and informing participants that they do not have to complete EMA surveys during these periods and that these surveys will not count against their compliance. Furthermore, participants are instructed not to complete EMA surveys when it would be unsafe or uncomfortable (eg, when driving or when a caregiver or peer is looking over their shoulder). Research assistants also inform participants that they will receive push notification reminders, SMS text messages, and telephone calls to facilitate their EMA compliance (greater detail on these procedures is provided in the subsections that follow). Finally, research assistants will inform participants that they will be in frequent contact with them to help facilitate EMA compliance and address any questions that may arise [104]. EMA compliance will be calculated in two ways: (1) total accountable EMA compliance (total number of EMA surveys completed/total number of EMA accountable surveys—accountable surveys refer to reports where an adolescent or caregiver could have completed the survey but did not; this will be the primary means of examining EMA compliance) and (2) total EMA compliance (total number of EMA surveys completed/total number of EMA surveys).

### EMA Burst Design and Content

#### Overview

Caregivers and adolescents will complete 9 weeks of EMA after their baseline study visit session over the course of three 21-day EMA burst periods (Figure 1). Figure 2 provides a detailed overview of the EMA procedures for each study day. The EMA protocol will consist of 2 assessment types, which are discussed in the following subsections.

#### Morning Reports

*Morning reports* will be interval-contingent recordings whereby adolescents and caregivers answer questions each morning about caregiving practices; adolescent internalizing and externalizing symptoms; and adolescent past-day alcohol, cannabis, and other substance use (refer to Multimedia Appendix 1 for a complete list of morning report items). Morning reports will be available each day from 4 AM to 11:59 PM. If a participant has not completed the morning report, they will receive push notifications at 11 AM and 3 PM asking them to complete their morning report. Participants will receive an SMS text message at 6:30 PM asking them to complete their morning report if it has not been completed yet. Morning reports were selected over evening reports to reduce substance use recall bias because most adolescent alcohol use occurs in the late evenings [105,106].

#### Random Prompts

*Random prompts* (signal-contingent reports) will occur at random intervals from 3 PM to 10 PM during the school week and from 11 AM to 10 PM during the weekend. Blocks will be delivered randomly in 140-minute intervals during weekdays and 165-minute intervals during weekends to yield 3 and 4 random prompts per day, respectively. These numbers of prompts have been found to be manageable for adolescents and caregivers [107]. Participants will be alerted to their random prompts through push notifications on their mobile phones, and the random prompts will be programmed to ensure that they do not occur within a 30-minute window of each other. Participants will receive a push notification asking them to complete the random prompt after 10 minutes if it has not been completed yet. If a participant does not complete the random prompt after 20 minutes, they will receive a text message asking them to complete the random prompt and notifying the participant that they have only 10 minutes left to complete the assessment. Considering that the overarching goal of the protocol is to study caregiving practices in the moment, the random prompts will be delivered to caregivers and adolescents at the same time and will be available for 30 minutes. A concern with a longer window to complete random prompts is that there could be a larger temporal lag between adolescent- and caregiver-reported behaviors (eg, a caregiver completes the random prompt immediately and then the adolescent completes the random prompt after 50 min).

Multimedia Appendix 1 contains a complete list of the Project MAPP EMA items. Caregivers and adolescents will be asked whether they had contact (eg, in person, via SMS text message, or on the telephone) with each other since the last random prompt and complete the measures of caregiver communication as well as general and alcohol-specific caregiving, assessing these behaviors since the last random prompt. Random prompts are commonly used to assess social interactions in EMA research [108-110]. Each random prompt will capture any substance use since the last report, the time use began and ended, and the amount consumed (Table 1). The random prompts will also capture adolescent treatment motivation, alcohol craving, affect, and contextual information (eg, who they are with).

### EMA Participant-Tracking Procedures

High levels of EMA compliance are essential to obtaining high-quality data that can rigorously answer the questions outlined in this protocol. The proposed study will implement the following procedures to maximize EMA compliance: (1) As noted in the Baseline Study Visit subsection, the dyads will be trained during the baseline visit by research assistants on using MetricWire and review the times when participants are unable to answer prompts and should not answer prompts (eg, when driving). (2) Research assistants will monitor the EMA compliance of all participants daily. (3) Participants will be contacted frequently through the MetricWire messaging feature or SMS text message, based on their preference, to receive EMA compliance feedback. (4) On the morning of the fourth day of each 7-day EMA week period, participants will be sent a message noting whether they are on pace to earn a US $10 bonus for maintaining a weekly compliance rate of ≥75%. For participants who are below the 75% threshold, they will be informed of the number of surveys they need to complete during the remainder of the week to earn the US $10 compliance bonus. (5) At the end of each study week, research assistants will reach out to participants by text message or telephone call, depending on participant preference, to review the participants’ EMA compliance from the last week, discuss missed surveys and whether there were any reasons for missed surveys, and inquire whether there were any issues that occurred with the EMA software. (6) Information from procedures 3 to 5 will be shared with all Project MAPP staff on an internal messaging service (eg, Google Chat or Slack). This will help ensure that the entire study team is aware of any emerging issues with EMA compliance and that plans can be discussed early on to address any compliance issues. In addition, this information will be reviewed at weekly Project MAPP study meetings.

### Mental Health Update Reports

Considering that aim 3 seeks to examine whether the use of family-focused therapy techniques are associated with changes in caregiving practices, Project MAPP requires a means of collecting information on what is occurring in weekly therapy sessions. The mental health update report will collect information, weekly, on the skills that adolescents and caregivers are learning in outpatient therapy. These reports will capture skills learned or practiced in therapy, caregiver involvement in the session, therapeutic alliance, and the degree to which caregivers support their adolescent’s mental health and treatment goals (refer to Multimedia Appendix 1 for a complete list of items). The skills that caregivers and adolescents learn or practice in session will be assessed using the Therapy Process Observation Coding Scale–Self-Reported Therapist Intervention Fidelity for Youth [111], modified for caregiver and adolescent self-reports. This measure captures adolescent-oriented skills (eg, emotion regulation, managing negative thinking, and behavioral activation) and caregiver and family-oriented skills (eg, communication skills, consistent rules, and consequences). When adolescents or caregivers report that an adolescent missed their weekly therapy session, they will complete the mental health update report, which will include specific items assessing why they missed their therapy visit along with most of the constructs assessed in the mental health update report for attended sessions (Multimedia Appendix 1).

Unfortunately, only approximately 4 in 10 individuals of any age who present to substance use services complete treatment [112]. This number may be lower for adolescents [113]; for example, whereas the average outpatient treatment protocol is 16.5 sessions, youth only attend 3.9 sessions on average [114]. Acknowledging the high dropout rates in adolescent outpatient treatment, this study will retain dyads where the adolescent drops out of treatment. If an adolescent or caregiver reports that the adolescent dropped out of treatment, they will complete a version of the mental health update report assessing the reasons for discontinuing treatment, alternative recovery supports that the adolescent is receiving, caregiver support of the adolescent’s mental health and substance use, the use of skills learned from therapy or other recovery supports, and experiences of minority stress (refer to Multimedia Appendix 1 for a complete list of items). Dyads where the adolescent successfully completes treatment will also complete the same version of the mental health update report but not be administered questions regarding the reasons for discontinuing treatment (Multimedia Appendix 1).

The mental health update report will be available every Saturday starting at 4 AM. Reminders for the mental health update will follow the same schedule as those for the morning reports. If the participant does not complete the mental health update on Saturday, the mental health update will be available to complete on Sunday.

### Statistical Analyses

#### Data Analytic Approach

The proposed EMA protocol will generate a large database with clustered data. The aims of Project MAPP require a data analytic approach that can accommodate the clustering of reports (level 1) within participants (level 2), the unique timing of reports for each participant in the study, and missingness. Aims 1 and 2 will be assessed using group iterative multiple model estimation (GIMME), an analytic approach that was designed for clustered data [115]. GIMME will be used to model contemporaneous and temporal associations between caregiving practices and adolescent treatment targets [115]. A benefit of GIMME over traditional multilevel modeling techniques for intensive longitudinal data is that it uses a data-driven technique to estimate person-specific networks consisting of both lagged and contemporaneous associations based on unified structural equation modeling. GIMME will be conducted using the R package *gimme* [116].

#### Descriptive Statistics

Descriptive statistics (eg, means and SDs) will be reported for all focal predictors and outcomes noted in aims 1 to 3. Intraclass correlation coefficients (ICCs) will be calculated for these variables to characterize the degree of variation across participants (ie, between-person variation) and across days or moments (ie, within-person variation) in these constructs. In addition, multilevel growth modeling, conducted separately for each focal predictor (eg, caregiver support) and outcome (eg, alcohol craving) will examine mean level change in caregiving behaviors and core treatment targets over the course of the study.

#### Examine Momentary Associations Between Caregiving and Core Treatment Targets (Aim 1)

##### Overview

Hypothesis 1 is that momentary *caregiver reports* of caregiver support, caregiver monitoring, substance use communication quality, and alcohol-specific caregiving practices will be positively associated with *adolescent reports* of motivation to reduce or stop drinking and positive affect while negatively associated with alcohol craving, alcohol use, and internalizing and externalizing symptoms. Momentary *caregiver reports* of caregiver-adolescent conflict will be negatively associated with *adolescent reports* of motivation to reduce or stop drinking and positive affect while positively associated with alcohol craving, alcohol use, and internalizing and externalizing symptoms. GIMME will be used to model contemporaneous and temporal associations between parenting practices and adolescent core treatment targets [115]. Default settings will predominantly be used across models. Specifically, we will allow autoregressive paths to be freely estimated; and the inference criteria for path pruning will use a Bonferroni correction of α=.05/N, where N is the number of individuals [117,118]. Data will be standardized within GIMME. GIMME uses full information maximum likelihood to account for observations with missing data.

##### GIMME 1: Positive Affect, Negative Affect, Alcohol Craving, Motivation to Reduce or Stop Drinking, and Alcohol Use Outcomes

Random prompts during the 9 weeks of EMA will be used to examine the contemporaneous and prospective associations of caregiver support, caregiver-adolescent conflict, caregiver monitoring, substance use communication quality, and alcohol-specific caregiving practices with positive affect, negative affect, alcohol craving, motivation to reduce or stop drinking, and alcohol use. Signal-contingent reports that occur after alcohol use each day will be excluded due to adolescent substance use altering parenting behaviors [87]. Although affect, craving, motivation, and use will serve as the central outcome variables, they will also predict caregiving behaviors in the model because GIMME estimates contemporaneous and lagged associations between all variables in the network. Bidirectional associations will not be included for alcohol-specific caregiving practices because GIMME currently does not support dichotomous outcome variables; therefore, we will be restricted to examining how the use of alcohol-specific caregiving practice (0=*practice not used* and 1=*practice used*) is associated with contemporaneous and future affect, craving, motivation to reduce or stop drinking, and alcohol use.

If there are a sufficient number of observations per participant, sensitivity analyses using the analytic approach in aim 2 (confirmatory subgroup GIMME [CS-GIMME] [119]; for details, refer to the Examine Changes in the Strength of the Associations Between Caregiving Practices and Core Treatment Targets Over the Course of Treatment [Aim 2] subsection) will compare these associations in reports before versus after adolescent alcohol use to understand how alcohol use may alter these relationships.

##### GIMME 2: Outcomes for Internalizing and Externalizing Symptoms

Morning reports from the 15 weeks of data collection (Figure 1) will be used to examine the contemporaneous and prospective associations of caregiver support, caregiver-adolescent conflict, caregiver monitoring, substance use communication quality, and alcohol caregiving practices with internalizing and externalizing symptoms. Caregiver support, caregiver-adolescent conflict, caregiver monitoring, substance use communication quality, and alcohol caregiving practices will be taken from the random prompts and averaged within day. These analyses will examine associations between caregiving practices and internalizing and externalizing symptoms within day and across days.

#### Examine Changes in the Strength of the Associations Between Caregiving Practices and Core Treatment Targets Over the Course of Treatment (Aim 2)

Hypothesis 2, aim 1, is that associations for caregiver support, caregiver monitoring, substance use communication quality, and alcohol-specific caregiving practices will become stronger over the course of AUD+CODs treatment as caregivers and adolescents learn and practice skills over the course of treatment. Aim 1 associations for caregiver-adolescent conflict will become weaker as caregivers and adolescents learn and practices skills to better manage conflict in the moment. We will use CS-GIMME [119], an extension of GIMME that can model subgroup-level associations within predefined subgroups (ie, EMA burst periods). Subgroups will be defined as EMA burst periods 1, 2, and 3. The CS-GIMME algorithm searches across EMA bursts (ie, group level) for contemporaneous and lagged associations that improve model fit for most of the individuals. Consistent with prior work, the subgroup-level cutoff (ie, in the 3 separate burst periods) will be 51% [120,121], meaning that paths will be included in subgroup networks if they improve fit for at least 51% of the subgroup. Finally, GIMME estimates effects for each individual. Conceptually, this information indicates whether the strength of the associations between caregiving and core treatment targets becomes more frequent (ie, more families demonstrate associations between parenting behaviors and core treatment targets) and whether these associations become stronger across treatment. CS-GIMME will be conducted separately for the 2 sets of outcomes outlined in aim 1. Of note, whereas CS-GIMME for model 1 in aim 1 will be based on burst periods, the CS-GIMME for model 2 in aim 1 will be based on 5-week periods (ie, study weeks 2-6, 7-11, and 12-16).

#### Examine Whether the Caregiver-Reported Use of Family-Focused Therapy Techniques Is Associated With Changes in Parenting Practices (Aim 3)

Hypothesis 3 is that caregiver reports of learning or practicing family focused therapy techniques in therapy will be prospectively positively associated with increases in caregiver support, caregiver monitoring, substance use communication quality, and alcohol-specific caregiving practices and with decreases in caregiver-adolescent conflict. Multilevel modeling will be used to predict caregiving practices using EMA data. Repeated observations (level 1) will be nested within participants (level 2). Caregiver support, caregiver-adolescent conflict, caregiver monitoring, substance use communication quality, and alcohol caregiving practices will be predicted by techniques learned or practiced in therapy from mental health reports. Caregiving practices will be averaged across therapy weeks (eg, caregiver support will be averaged between therapy sessions X and X+1) to allow for prospective associations between skill receipt and subsequent use. Separate multilevel models will be conducted for each caregiving practice.

#### Power Calculations

##### Aims 1 and 2

The ability to detect effects in GIMME analysis is a function of the number of time point numbers, rather than sample size. Simulation studies have found that GIMME can adequately detect signal from noise and recover accurate group and individual models when there are a minimum of 10 participants and 60 time points (advisable) per person [118,122]. Recent work suggests that the direction of associations is best recovered in GIMME when autoregressive parameters are strong and when there are 100 observations per person [123]. Over the course of the 9 weeks of EMA, there will be 207 random prompts and 112 morning reports. For random prompts, participants would have to complete 29% (60/207) and 48.3% (100/207) of their assessments to reach recommended observations per person for GIMME. For morning report–based analyses (ie, examining internalizing and externalizing symptom outcomes), participants would have to complete 53.6% (60/112) and 89.3% (100/112) of their morning reports to reach recommended observations per person for GIMME.

##### Aim 3

Assuming a power level of 0.8, a small or medium ICC, and 75% compliance with mental health update reports, the minimum detectable effect sizes for level-1 direct effects in the multilevel model would be 0.15 if the participant retention rate is 93% and 0.17 if the retention rate is 67% [124]. Assuming a power level of 0.8, a small ICC, and 75% compliance with mental health update reports, the minimum detectable effect sizes for level-2 direct effects would be 0.43 with a 93% retention rate and 0.49 with a 67% retention rate [124]. For a medium ICC, the minimum detectable effect sizes for level-2 direct effects would be 0.36 with a 93% retention rate and 0.41 with a 67% retention rate. Considering that the direct effects of interest for aim 3 are level-1 effects, the proposed study would be powered to detect small effects between therapy techniques and the use of caregiving practices.

## Results

### Pilot Work

As part of a National Institute on Alcohol Abuse and Alcoholism K99 award (K99AA030030), a pilot trial was conducted to assess the acceptability and feasibility of conducting a 7-week EMA study with caregiver-adolescent dyads, in which the adolescent is receiving AUD+CODs treatment in an intensive outpatient treatment program. The acceptability and feasibility benchmarks for the K99 phase are presented in Table 2. Whereas some core benchmarks were met during the K99 pilot study (ie, retention rate ≥80% for caregivers), most of the benchmarks were not met. These included a targeted enrollment rate of ≥75% versus the actual dyad enrollment rate of 62% (16/26), retaining 75% (12/16) of the adolescents versus an 80% target retention rate, EMA random prompt completion rate of ≥80% versus adolescents completing 47.6% (772/1622) and caregivers completing 70.76% (1331/1881) of the random prompts, and a morning report compliance rate of ≥80% versus adolescents completing 55.5% (361/651) and caregivers completing 91.3% (601/658) of the morning reports. The proposed study, which would be funded through the R00 portion of this National Institute on Alcohol Abuse and Alcoholism Pathways to Independence R00 award, contains modifications based on the information learned from the K99 phase, which are discussed in detail in the Methods section (Table 2; Multimedia Appendix 2).

**Table 2:** K99 pilot study benchmarks and corresponding R00 proposed changes.

K99 benchmark	Outcome	Proposed R00 protocol modification
Target enrollment rate of ≥75%	Of the 26 eligible adolescent-caregiver dyads, 16 (62%) were enrolled	Considering that 20.5% of the adolescents did not participate due to citing the severity of their co-occurring symptoms or they went to a higher level of care before their baseline study visit, the R00 phase will recruit from community outpatient substance use clinics, a lower level of care Whereas the K99 phase only recruited from a single clinic, the R00 phase will recruit from 5 community clinics; the larger potential participant pool across the 5 community clinics will facilitate meeting the recruitment goals of the R00 phase even if enrollment rates fall below the threshold of ≥75% of eligible dyads
Retain ≥80% of the adolescents and caregivers from the baseline assessment through the completion of the 7-week EMA^a^ protocol and discharge assessment	100% of the dyads were retained during the 7-week EMA period; 75% (12/16) of the adolescents and 81% (13/16) of the caregivers completed the discharge session	Considering that 50% of the adolescents did not complete the discharge session because they were being provided a higher level of care (ie, inpatient hospitalization or residential treatment program), the R00 phase will recruit from community outpatient substance use clinics, a lower level of care
Completion of ≥80% of the random prompts	Across the 7-week EMA period, adolescents completed 47.6% (772/1622) and caregivers completed 70.76% (1331/1881) of the random prompts	Adolescent EMA data linearly declined by week (refer to Multimedia Appendix 2), with a large decrease in compliance between weeks 3 and 4; for the R00 phase, EMA will be administered through a burst designb wherein dyads will complete 3 weeks of EMA, have a 3-week break, complete 3 weeks of EMA, have a 3-week break, and then complete 3 weeks of EMA To improve compliance, EMA random prompts will only be sent to adolescents and their caregivers from 3 PM to 10 PM during weekdays to avoid prompts being delivered during school hours (this modification will lead to 1 less random prompt each day during weekdays) The compensation structure will be modified in the R00 phase; in the K99 phase, participants were compensated when they completed 3 out of 5 surveys per day, whereas in the R00 phase, participants will be compensated for each survey they complete (moving to this compensation schedule will incentivize participants to complete surveys even when they can only complete 1 or 2 prompts per day)
Completion of ≥80% of the morning reports	Adolescents completed 55.5% (361/651) and caregivers completed 91.3% (601/658) of the morning reports	As noted in the modifications based on random prompt compliance, participants were only compensated if they completed 3 out of 5 surveys per day; if participants were unable to answer 3 random prompts (eg, because they were driving or were at school), they may not have completed their morning report because there was no financial incentive to do so; to address this, the R00 phase will compensate participants for each survey they complete, including morning reports

^a^EMA: ecological momentary assessment.

^b^Whereas intensive outpatient programs have more limited and standardized treatment durations, community outpatient clinics vary in their length of treatment. Whereas some of the partnering R00 clinics deliver the 12- to 14-session manualized treatment—the adolescent community reinforcement approach (A-CRA)—others see their adolescent clients for a year or longer. In addition to using a burst design to help increase random prompt compliance, a 15-week burst design EMA study will fully capture the length of treatment for clinics delivering A-CRA and to sample a sufficient period of treatment (ie, almost 4 months) to appropriately test the aims of the R00 phase.

### Proposed Study Timeline

No data have been collected for the proposed study. Data collection is anticipated to begin in December of 2024. Project MAPP will take place over the course of 3 years. Study startup tasks (eg, training study staff) will occur during months 1 to 3, and data collection will occur during months 4 to 30. On the basis of this recruitment time frame, the study will need to enroll approximately 3 dyads per month to meet the recruitment goal of 75 dyads. Data checks to ensure the quality of the data will be performed weekly throughout data collection as will data cleaning procedures. Beginning in month 31, by which time most of the data collection will have occurred, data preparation and analysis will be performed for the primary aims of Project MAPP.

## Discussion

### Summary

Project MAPP seeks to advance our knowledge of how caregiver involvement in AUD+CODs treatment improves outcomes by examining (1) momentary associations between caregiving practices and core treatment targets (eg, motivation to reduce or stop drinking and alcohol craving), (2) changes in the strength of the associations in aim 1 to understand whether caregiving practices become more effective over the course of therapy, and (3) whether learning and practicing family-focused therapy techniques in session are associated with changes in the use of caregiving practices in real time in daily life outside of therapy. Information from this study will hopefully aid clinical practice by informing which caregiving practices positively change adolescent core treatment targets on a more refined and clinically aligned timescale. In contrast to existing caregiving research that indicates that greater monitoring before treatment is associated with improved outcomes [125], the proposed study will be able to determine whether caregiver use of monitoring strategies in the moment protects their children from using substances. Data from this study will be on a timescale most relevant to caregivers and clinicians who want to know what caregivers can do during a specific moment in time to promote well-being and mitigate risk for their teens when in treatment [126,127].

### Additional Questions of Interest

In addition to testing the primary aims of Project MAPP, we intend to use the data from this study to advance knowledge on associations across multiple timescales (eg, the association between learning or practicing skills and then using them outside of therapy). Theoretical and empirical papers in psychological science have raised awareness of the importance of timescale [54,128]. Aims 1 to 3 of Project MAPP focus on momentary (aims 1 and 2) and proximal (ie, weekly; aim 3) associations. It remains an open empirical question as to whether these are the most appropriate timescales to better understand caregiving processes during treatment; for example, aim 3 will examine whether caregiver skills learned in therapy are associated with the use of caregiving practices in the week after therapy. Alternative timescales (eg, lags of half a week, 2 weeks, 1 month, and the entire study period) may better capture the relationship between learning or practicing skills in therapy and implementing them outside of therapy.

Aims 1 to 3 emphasize the unidirectional role of caregiving behaviors on adolescent core treatment outcomes. A large body of research demonstrates that caregiving and adolescent behavior influence one another reciprocally across time [129-131]. Changes in adolescent core treatment outcomes during AUD+CODs treatment may be a function of improvements in adolescent behavior, which result in improved caregiving behaviors. In turn, these caregiving improvements facilitate positive changes in adolescent treatment outcomes. Furthermore, caregiver involvement in treatment may facilitate treatment outcomes by enhancing the effectiveness of skills that adolescents learn during therapy. Learning or practicing specific skills in therapy, such as emotion regulation strategies, may be more strongly associated with reduced mental health symptoms among adolescents whose caregivers monitor their behavior or are responsive to them. Project MAPP is designed to test alternative mechanisms of positive outcomes, such as these examples, in addition to the primary aims of the study.

### Limitations

Although Project MAPP represents an important extension to caregiving research during AUD+CODs treatment, the proposed study will still contain several limitations. First, the study will recruit only a single caregiver per adolescent. Caregiving research has demonstrated unique effects of separate caregivers (eg, unique effects of mothers vs fathers [132]). By not including all caregivers responsible for the adolescent, Project MAPP will miss important information regarding what some caregivers are doing during AUD+CODs treatment. Second, caregivers and adolescents will be repeatedly asked questions about caregiving practices, skill use, treatment motivation, and substance use. There is the possibility for reactivity—that repeated assessments systematically alter caregiver and adolescent behaviors or attitudes [133,134]. Without the inclusion of a control condition (ie, a group of dyads in which the adolescents are receiving treatment who do not receive EMA), Project MAPP will not be able to determine the extent of reactivity to the EMA burst design protocol. Third, given that adolescents and their caregivers will be participating in outpatient treatment, which can last more than a year, Project MAPP will not be able to sample all of an adolescent’s time in AUD+CODs treatment. Finally, there are many caregiving practices associated with adolescent substance use [39]. Capturing all these practices via EMA would not be feasible. The selection of caregiving practices was based on the core elements of caregiver-involved interventions for adolescents [8,32] and EMA caregiving research from community samples [53,135].

## Appendix

Multimedia Appendix 1 Ecological momentary assessment items for Project Momentary Assessment of Parenting Practices.

Multimedia Appendix 2 Pilot study ecological momentary assessment compliance by study week.

## Abbreviations

A-CRA: adolescent community reinforcement approach
AUD: alcohol use disorder
AUD+COD: alcohol use disorder and co-occurring disorder
CS-GIMME: confirmatory subgroup group iterative multiple model estimation
EMA: ecological momentary assessment
GIMME: group iterative multiple model estimation
HIPAA: Health Insurance Portability and Accountability Act
ICC: intraclass correlation coefficient
MAPP: Momentary Assessment of Parenting Practices
TLFB: timeline followback

## Data Availability

All data collected from Project Momentary Assessment of Parenting Practices will be available through the National Institute on Alcohol Abuse and Alcoholism data archive.
