# Supplementary material for: Examining Caregiver Practices During Adolescent Outpatient Alcohol Use and Co-Occurring Mental Health Treatment: Protocol for a Dyadic Ecological Momentary Assessment Study
Source: JMIR Res Protoc. 2024 Dec 20;13:e63399. doi: 10.2196/63399 (PMC11702013; doi:10.2196/63399)
Supplement: Multimedia Appendix 1 [file resprot_v13i1e63399_app1.docx]

**Supplemental materials**

Contents

[Morning Report - Adolescent 2](#_Toc165703511)

[Morning Report - Caregiver 8](#_Toc165703512)

[Random Prompt - Adolescent 12](#_Toc165703513)

[Random Prompt – Caregiver 17](#_Toc165703514)

[Mental Health Update Report - Adolescent (therapy session) 22](#_Toc165703515)

[Mental Health Update Report - Adolescent (Did Not Have Therapy/Missed Session) 26](#_Toc165703516)

[Mental Health Update Report – Adolescent (quit or finished therapy) 30](#_Toc165703517)

[Mental Health Update Report – Caregiver (therapy session) 33](#_Toc165703518)

[Mental Health Update Report – Caregiver (Did Not Have Therapy/Missed Session) 38](#_Toc165703519)

[Mental Health Update Report – Caregiver (Quit or Finished Therapy) 42](#_Toc165703520)

[Mental Health Update Report – Caregiver (not sure if child had a therapy session) 45](#_Toc165703521)

**Morning Report Instructions, Timing Parameters, and General Functions**

1. Participants are instructed to complete the morning report (MR) once a day.
2. Morning report triggers are “date and time” triggered and will go off every study day (15 weeks) at a fixed time of 4 AM.
3. The trigger will begin on the day the participant enrolls.
4. The MR option is removed from the screen once the MR is completed each day. The MR will reappear at 4 AM the following day (without any push function).
5. If a participant has not completed the morning report, they will receive push notifications at 11am and 3pm asking them to complete their morning report. Participants will receive a text message at 6:30pm asking them to complete their morning report if it has not been completed yet. If the participant fails to respond to the notifications, the MR will remain available on the home screen until 11:59 PM that day.

# Morning Report - Adolescent

| **ITEM** | **RESPONSE OPTION** | | | | | | | | | | | |
| --- | --- | --- | --- | --- | --- | --- | --- | --- | --- | --- | --- | --- |
| 1. What time did you go to sleep last night? | (Hour and minute response option) | | | | | | | | | | | |
| 1. What time did you wake up today? | (Hour and minute response option) | | | | | | | | | | | |
| 1. How long did it take you to fall asleep? | (Hour and minute response option) | | | | | | | | | | | |
| 1. How would you rate the quality of your sleep last night? | (1) poor to (5) very good | | | | | | | | | | | |
| 1. Check the box besides all the types of substance you used yesterday. | □ None  □ Alcohol  □ Marijuana  □ Nicotine  □ Energy Drink (Red bull, etc.)  □ Inhalant  □ Opiates (Vicodin, etc.)  □ Non-prescribed stimulant (Adderall, etc.)  □ Stimulant as prescribed (Adderall, etc.)  □ Mushrooms  □ Other | | | | | | | | | | | |
| **Note**: Q6-Q11 will only be administered if a participant endorses alcohol use in Q5. | | | | | | | | | | | | |
| 1. Check the box beside all the types of alcohol you drank yesterday? | □ Beer (Bud Light, etc.)  □ Malt Liquor (Colt 45, etc.)  □ Liquor (straight or mixed)  □ Wine (Merlot, etc.)  □ Wine Cooler or hard seltzer (Boone’s, etc.)  □ Fortified Wine (Mad Dog 20/20, etc.)  □ I did not drink yesterday | | | | | | | | | | | |
| 1. Enter the number of standard drinks or ounces of [pipe alcoholic beverage from Q6] you drank yesterday. |  | | | | | | | | | | | |
| 1. You entered [show amount recorded] standard drinks or [show amount] ounces of alcohol. Is this correct? | Yes | | | | | | No | | | | | |
| 1. Who were you with when you were drinking? | □ No one  □ Friend(s) I drink with  □ Friend(s) I don’t drink with  □ Romantic partner I drink with  □ Romantic partner I don’t drink with  □ Mother  □ Father  □ Brother(s) or sister(s)  □ Other relative(s)  □ Friend’s parents  □ Coworkers  □ Other(s) | | | | | | | | | | | |
| 1. Did you drink more than planned yesterday? | 0=Not at all | | | | | | 10=Extremely | | | | | |
|  | 0 | 1 | 2 | 3 | 4 | 5 | | 6 | 7 | 8 | 9 | 10 |
| 1. In response to my drinking yesterday, my parent or caregiver (check all that apply) | □ My parent or caregiver did not know that I drank  □ Yelled at me in disapproval  □ Talked with me about why I shouldn’t drink  □ Talked with me about why I drank alcohol  □ Talked with me about how my drinking made them feel  □ Took away privileges (e.g., electronics)  □ Grounded me  □ Did not speak to me or ignored me  □ Offered me a reward not to drink  □ Did nothing | | | | | | | | | | | |
| **Note**: Q12-Q15 will only be administered if a participant endorses cannabis use in Q5. | | | | | | | | | | | | |
| 1. Check the box besides all the types of marijuana you used yesterday. | □ Smoked (joint, pipe bong, etc.)  □ Vaped (liquid, wax, dabs, shatter, etc.)  □ Edibles (baked goods, candies, gummies, etc.)  □ Beverages containing marijuana  □ Applied to skin (oils, tinctures, etc.)  □ Other | | | | | | | | | | | |
| 1. How high did you feel yesterday? | 0 (not at all) to 100 (highest I have ever felt) | | | | | | | | | | | |
| 1. Did you use marijuana more than planned yesterday? | 0=Not at all | | | | | | 10=Extremely | | | | | |
|  | 0 | 1 | 2 | 3 | 4 | 5 | | 6 | 7 | 8 | 9 | 10 |
| 1. In response to my marijuana use yesterday, my parent or caregiver (check all that apply) | □ My parent or caregiver did not know that I used marijuana  □ Yelled at me in disapproval  □ Talked with me about why I shouldn’t use marijuana  □ Talked with me about why I used marijuana  □ Talked with me about how my marijuana use made them feel  □ Took away privileges (e.g., electronics)  □ Grounded me  □ Did not speak to me or ignored me  □ Offered me a reward not to use marijuana  □ Did nothing | | | | | | | | | | | |
| **Note**: Q16-Q18 will only be administered if a participant endorses nicotine use in Q5. | | | | | | | | | | | | |
| 1. Check the box besides all the types of nicotine you used yesterday. | □ E-cigarettes/vapes  □ Cigarettes  □ Cigars  □ Hookahs  □ Smokeless tobacco  □ Nicotine pouches  □ Other | | | | | | | | | | | |
| 1. Did you use nicotine more than planned yesterday? | 0=Not at all | | | | | | 10=Extremely | | | | | |
|  | 0 | 1 | 2 | 3 | 4 | 5 | | 6 | 7 | 8 | 9 | 10 |
| 1. In response to my nicotine use yesterday, my parent or caregiver (check all that apply) | □ My parent or caregiver did not know that I used nicotine  □ Yelled at me in disapproval  □ Talked with me about why I shouldn’t use nicotine  □ Talked with me about why I used nicotine  □ Talked with me about how my nicotine use made them feel  □ Took away privileges (e.g., electronics)  □ Grounded me  □ Gave me the silent treatment  □ Offered me a reward not to use nicotine  □ Did nothing | | | | | | | | | | | |
| **Note**: Q19 will only be administered if a participant endorses a substance other than alcohol, nicotine, or cannabis in Q5. | | | | | | | | | | | | |
| 1. In response to my [pipe substance other than alcohol, marijuana, or nicotine selected in Question 1], my parent or caregiver (check all that apply) | □ My parent or caregiver did not know that I used [pipe substance]  □ Yelled at me in disapproval  □ Talked with me about why I shouldn’t use [pipe substance]  □ Talked with me about why I used [pipe substance]  □ Talked with me about how my [pipe substance] use made them feel  □ Took away privileges (e.g., electronics)  □ Grounded me  □ Gave me the silent treatment  □ Offered me a reward not to use [pipe substance]  □ Did nothing | | | | | | | | | | | |
| **NOTE**: If a participant answers “no” to prior day alcohol use, they will receive the following items (S1-S6). | | | | | | | | | | | | |
| S1. How much time did you spend on social media yesterday? | (enter time in hours and minutes) | | | | | | | | | | | |
| S2. How many of the posts/stories/snaps/ direct messages you viewed on social media yesterday had any alcohol-related content (e.g., picture, reference to alcohol, video of people drinking)? | 0 (none) to 10 (all) | | | | | | | | | | | |
| S3. How many of the posts/stories/snaps/ direct messages you viewed on social media yesterday had any marijuana-related content (e.g., picture, reference to marijuana, video of people smoking)? | 0 (none) to 10 (all) | | | | | | | | | | | |
| S4. How many of the posts/stories/snaps/ direct messages you viewed on social media yesterday had any mental health-related content? | 0 (none) to 10 (all) | | | | | | | | | | | |
| S5. How many of the posts/stories/snaps/ direct messages you read on social media yesterday had any content related to substance use treatment or recovery (e.g., video of people in recovery, references to cutting down substance use)? | 0 (none) to 10 (all) | | | | | | | | | | | |
| S6. Yesterday, what content did you post to social media? (check all the apply) | □ Alcohol-related  □ Marijuana-related  □ Nicotine-related  □ Mental health-related  □ Substance use treatment/recovery related  □ None of the above | | | | | | | | | | | |
| 1. Check the box beside all the substances you have or could get today. | □ None  □ Alcohol  □ Marijuana  □ Nicotine  □ Energy Drink (Red bull, etc.)  □ Inhalant  □ Opiates (Vicodin, etc.)  □ Non-prescribed stimulant (Adderall, etc.)  □ Stimulant as prescribed (Adderall, etc.)  □ Mushrooms  □ Other | | | | | | | | | | | |
| 1. Do you plan to drink today? | 0=Definitely Not 5=Undecided 10=Definitely, Yes | | | | | | | | | | | |
|  | 0 | 1 | 2 | 3 | 4 | 5 | | 6 | 7 | 8 | 9 | 10 |
| 1. Do you plan to use marijuana today? | 0=Definitely Not 5=Undecided 10=Definitely, Yes | | | | | | | | | | | |
|  | 0 | 1 | 2 | 3 | 4 | 5 | | 6 | 7 | 8 | 9 | 10 |
| 1. Do you plan to use nicotine today? | 0=Definitely Not 5=Undecided 10=Definitely, Yes | | | | | | | | | | | |
|  | 0 | 1 | 2 | 3 | 4 | 5 | | 6 | 7 | 8 | 9 | 10 |
| 1. How much time did you and your parent or caregiver spend communicating (in person, over the phone, text-messaging, social media) yesterday? | □ None  □ A few minutes  □ 10 to 15 minutes  □ 20 to 30 minutes  □ 1 hour  □ 2 hours  □ 3 hours or more | | | | | | | | | | | |
| 1. How much did you and your parent or caregiver argue, disagree, or get upset with each other yesterday? | 0 - Not at all  1 - A little  2 - Somewhat  3 - Very much  4 - Extremely | | | | | | | | | | | |
| 1. Yesterday, you … (check all that apply) | □ Spent time with your friends  □ Talked to someone by phone, text, or online  □ Had school or other activities  □ Had homework/papers/tests  □ Had free time  □ Watched TV or videos on the internet  □ Spent time online (e.g., video games, IM, websites)  □ Went on social media  □ None of the above | | | | | | | | | | | |
| **NOTE**: Some Q27 responses will be based on responses to Q26. Regardless of their answers to Q26, all participants will be asked in Q27 “About how you are doing with your friends,” and “About your substance use.” If a participant endorses “Had school or other activities,” “Had free time,” "Had homework/paper/tests,” “Watched TV or videos on the internet,” “Spent time online,” or “Went on social media,” they will answer question about their caregivers knowledge of these specific activities in Q27. | | | | | | | | | | | | |
| 1. Yesterday, your parent or caregiver knew (check all that apply) | □ About how you are doing with your friends  □ How you are doing in school or activities  □ What you did during your free time  □ What homework/papers/tests you had  □ Where and what you did after school  □ What you watched on TV or the internet  □ What you did online (video games, IM, websites)  □ What you did on social media  □ Who you talked to (including online, phone, text)  □ About your substance use  □ None of the above | | | | | | | | | | | |
| 1. Yesterday, you told your parent or caregiver without them asking (check all that apply) | □ About your friends  □ What happened or how you are doing in school or activities  □ What you did during your free time  □ What homework, papers, or tests you had  □ Where you went and what you did after school  □ What you watched on TV or the internet  □ What you did online (video games, IM, websites)  □ What you did on social media  □ Who you talked to (including online, phone, text)  □ About your substance use  □ None of the above | | | | | | | | | | | |
| 1. Yesterday, your parent or caregiver asked you (check all that apply) | □ About your friends  □ What happened or how you are doing at school or activities  □ What you did during your free time  □ What homework, papers, or tests you had  □ Where you went and what you did after school  □ What you watched on TV or the internet  □ What you watched and posted on social media  □ What you did online (video games, IM, websites)  □ Who you talked to (including online, phone, text)  □ About your substance use  □ None of the above | | | | | | | | | | | |
| **NOTE**: Question 30 will be administered if an adolescent did not endorse talking with their parent or caregiver about substance use yesterday (did not endorse “about their substance use” in Questions 28 or 29). Question 30b will be administered if an adolescent notes that their parent or caregiver spoke to them or they spoke to their parent or caregiver about substance use yesterday (Questions 28 or 29 “about their substance use).” If an adolescent endorses any response other than “Did nothing” for Questions 11, 15, 18, or 19, they will not complete Questions 30-30b and 31 and directly be asked question 32. | | | | | | | | | | | | |
| 1. Yesterday, did you talk to your parent or caregiver about (select all that apply) | □ Alcohol  □ Marijuana  □ Nicotine  □ Inhalant  □ Opiates (Vicodin, etc.)  □ Non-prescribed stimulant (Adderall, etc.)  □ Mushrooms  □ Other substance  □ I did not talk to my caregiver about substance use | | | | | | | | | | | |
| **NOTE**: If a participant selects that they did not talk to their caregiver about substance use they will proceed to Q35. | | | | | | | | | | | | |
| 30b. Yesterday, what substance did you talk to your parent or caregiver about (check all that apply) | □ Alcohol  □ Marijuana  □ Nicotine  □ Inhalant  □ Opiates (Vicodin, etc.)  □ Non-prescribed stimulant (Adderall, etc.)  □ Mushrooms  □ Other substance | | | | | | | | | | | |
| **NOTE**: Question 31 will only be administered if an adolescent selects “other substance” for Question 30 or 30b. | | | | | | | | | | | | |
| 1. What substance did you talk to your parent or caregiver about? | Open response | | | | | | | | | | | |
| 1. When my parent or caregiver and I spoke about my substance use yesterday, I felt UNDERSTOOD. | Not at all (0) – Very Much (5) | | | | | | | | | | | |
| 1. When my parent or caregiver and I spoke about my substance use yesterday, I felt COMFORTABLE. | Not at all (0) – Very Much (5) | | | | | | | | | | | |
| 1. When my caregiver and I spoke about my substance use yesterday, we easily discussed our OPINIONS. | Not at all (0) – Very Much (5) | | | | | | | | | | | |
| 1. Yesterday, I felt ANXIOUS | Not at all (0) to Very Much (7) | | | | | | | | | | | |
| 1. Yesterday, I felt DEPRESSED | Not at all (0) to Very Much (7) | | | | | | | | | | | |
| 1. Yesterday, I felt MY MOOD WAS UP AND DOWN |  | | | | | | | | | | | |
| 1. Yesterday, I felt WORRIED ABOUT BEING ABANDONED | Not at all (0) to Very Much (7) | | | | | | | | | | | |
| 1. Yesterday, I felt LITTLE INTEREST IN DOING MUCH OF ANYTHING | Not at all (0) to Very Much (7) | | | | | | | | | | | |
| 1. Yesterday, I felt LIKE I WANTED TO HURT SOMEONE | Not at all (0) to Very Much (7) | | | | | | | | | | | |
| 1. Yesterday, I felt LIKE I DIDN’T WANT TO BE AROUND OTHER PEOPLE | Not at all (0) to Very Much (7) | | | | | | | | | | | |
| 1. Yesterday, I felt LIKE MY RELATIONSHIPS ARE EMPTY | Not at all (0) to Very Much (7) | | | | | | | | | | | |
| 1. Yesterday, I acted ON MY EMOTIONS | Not at all (0) to Very Much (7) | | | | | | | | | | | |
| 1. Yesterday, I acted IMPULSIVELY | Not at all (0) to Very Much (7) | | | | | | | | | | | |
| 1. Yesterday, I acted ON IMPULSE WHILE FEELING UPSET | Not at all (0) to Very Much (7) | | | | | | | | | | | |
| 1. Yesterday, I acted AGGRESSIVELY TOWARD SOMEONE | Not at all (0) to Very Much (7) | | | | | | | | | | | |
| 1. Yesterday, I acted IRRESPONSIBLY | Not at all (0) to Very Much (7) | | | | | | | | | | | |
| 1. Yesterday, I SAID SOMETHING OFFENSIVE TO SOMEONE | Not at all (0) to Very Much (7) | | | | | | | | | | | |
| 1. Yesterday, I DID SOMETHING DANGEROUS TO SOMEONE | Not at all (0) to Very Much (7) | | | | | | | | | | | |
| 1. Yesterday, I LOST MY TEMPER | Not at all (0) to Very Much (7) | | | | | | | | | | | |

**Shortest Possible Length**: 38 items

**Longest Possible Length**: 49 items

**Morning Report Instructions, Timing Parameters, and General Functions**

1. Participants are instructed to complete the morning report (MR) once a day.
2. The MR option is removed from the screen once the MR is completed each day. The MR will reappear at 4 AM the following day (without any push function).
3. Morning report triggers are “date and time” triggered and will go off every study day (15 weeks) at a fixed time of 4 AM.
4. The trigger will begin on the day the participant enrolls.
5. If a participant has not completed the morning report, they will receive push notifications at 11am and 3pm asking them to complete their morning report. Participants will receive a text message at 6:30pm asking them to complete their morning report if it has not been completed yet. If the participant fails to respond to the notifications, the MR will remain available on the home screen until 11:59 PM that day.

# Morning Report - Caregiver

| **ITEM** | **RESPONSE OPTION** |
| --- | --- |
| 1. Check the box besides all the types of substance your child used yesterday. | □ None  □ Alcohol  □ Marijuana  □ Nicotine  □ Energy Drink (Red bull, etc.)  □ Inhalant  □ Opiates (Vicodin, etc.)  □ Non-prescribed stimulant (Adderall, etc.)  □ Stimulant as prescribed (Adderall, etc.)  □ Mushrooms  □ Other |
| **NOTE**: Q2 will be administered if a caregiver selects “alcohol” in Q1. Q3 will be administered if a caregiver selects “marijuana” in Q1. Q4 will be administered in a caregiver selects “nicotine” in Q1. Q5 will be administered if a caregiver selects any substance in Q1 other than alcohol, marijuana, or nicotine. | |
| 1. In response to my child’s drinking yesterday, I (check all that apply) | □ Yelled at them in disapproval  □ Talked with them about why they shouldn’t drink  □ Talked with them about why they drank alcohol  □ Talked with them about how their drinking made me feel  □ Took away privileges (e.g., electronics)  □ Grounded them  □ Gave them the silent treatment  □ Offered them a reward not to drink  □ Did nothing |
| 1. In response to my child’s marijuana use yesterday, I (select all that apply) | □ Yelled at them in disapproval  □ Talked with them about why they shouldn’t use marijuana  □ Talked with them about why they used marijuana  □ Talked with them about how their marijuana use made me feel  □ Took away privileges (e.g., electronics)  □ Grounded them  □ Gave them the silent treatment  □ Offered them a reward not to use marijuana  □ Did nothing |
| 1. In response to my child’s nicotine use yesterday, I (select all that apply) | □ Yelled at them in disapproval  □ Talked with them about why they shouldn’t use nicotine  □ Talked with them about why they used nicotine  □ Talked with them about how their nicotine use made me feel  □ Took away privileges (e.g., electronics)  □ Grounded them  □ Gave them the silent treatment  □ Offered them a reward not to use nicotine  □ Did nothing |
| 1. In response to my child’s [pipe substance other than alcohol, marijuana, or nicotine selected in Question 1], I (select all that apply) | □ Yelled at them in disapproval  □ Talked with them about why they shouldn’t use [pipe substance]  □ Talked with them about why they used [pipe substance]  □ Talked with them about how their [pipe substance] use made me feel  □ Took away privileges (e.g., electronics)  □ Grounded them  □ Gave them the silent treatment  □ Offered them a reward not to use [pipe substance]  □ Did nothing |
| **NOTE**: All participants will continue with Q6. | |
| 1. Check the box besides all the types of substance you used yesterday. | □ None  □ Alcohol  □ Marijuana  □ Nicotine  □ Energy Drink (Red bull, etc.)  □ Inhalant  □ Opiates (Vicodin, etc.)  □ Non-prescribed stimulant (Adderall, etc.)  □ Stimulant as prescribed (Adderall, etc.)  □ Mushrooms  □ Other |
| 1. How much time did you and your child spend communicating (in person, over the phone, text-messaging, social media) yesterday? | □ None  □ A few minutes  □ 10 to 15 minutes  □ 20 to 30 minutes  □ 1 hour  □ 2 hours  □ 3 hours or more |
| 1. How much did you and your child argue, disagree, or get upset with each other yesterday? | 0 - Not at all  1 - A little  2 - Somewhat  3 - Very much  4 - Extremely |
| 1. Yesterday, your child … (check all that apply) | □ Spent time with their friends  □ Talked to someone by phone, text, or online  □ Had school or other activities  □ Had homework/papers/tests  □ Had free time  □ Watched TV or videos on the internet  □ Spent time online (e.g., video games, IM, websites)  □ Went on social media  □ None of the above |
| **NOTE**: Some Q10 responses will be based on responses to Q9. Regardless of their answers to Q9, all participants will be asked in Q10 “How your child is doing with their friends,” and “About your child’s substance use.” If a participant endorses “Had school or other activities,” “Had free time,” "Had homework/paper/tests,” “Watched TV or videos on the internet,” “Spent time online,” or “Went on social media,” they will answer question about their knowledge of these specific activities in Q10. | |
| 1. Yesterday, you knew (check all that apply) | □ How your child was doing with their friends  □ How your child was doing in school or activities  □ What your child did during free time  □ What homework/papers/tests your child had  □ Where and what your child did after school  □ What your child watched on TV or the internet (e.g., YouTube)  □ What your child did online (video games, IM, websites)  □ What your child did on social media  □ Who your child talked to (including online, phone, text)  □ About your child’s substance use  □ None of the above |
| 1. Yesterday, your child told you, without you asking (check all that apply) | □ About their friends  □ How they are doing in school or activities  □ What they did during free time  □ What homework/papers/tests they had  □ Where and what they did after school  □ What they watched on TV or the internet (e.g., YouTube)  □ What they did online (video games, IM, websites)  □ What they did on social media  □ Who they talked to (including online, phone, text)  □ About their substance use  □ None of the above |
| 1. Yesterday, you asked your child | □ About their friends  □ How they are doing in school or activities  □ What they did during free time  □ What homework/papers/tests they had  □ Where and what they did after school  □ What they watched on TV or the internet (e.g., YouTube)  □ What they did online (video games, IM, websites)  □ What they did on social media  □ Who they talked to (including online, phone, text)  □ About their substance use  □ None of the above |
| **NOTE**: Q13 will be administered if a caregiver did not endorse talking with their child about substance use yesterday (did not endorse “about their substance use” in Q11 or Q12). Q14 will be administered if a caregiver notes that their child spoke to them or they spoke to their child about substance use yesterday (Q10 or Q11 “about their substance use.” Q15 will only be administered if a caregiver selects “other substance” for Q13 or Q14. If a caregiver endorses any response other than “Did nothing” for Q2-5, they will not complete Q16-18 and directly be asked Q20. | |
| 1. Yesterday, did you talk to your child about (check all that apply) | □ Alcohol  □ Marijuana  □ Nicotine  □ Inhalant  □ Opiates (Vicodin, etc.)  □ Non-prescribed stimulant (Adderall, etc.)  □ Mushrooms  □ Other substance  □ I did not talk to my child about substance use |
| 1. Yesterday, what substance did you talk to your child about (check all that apply) | □ Alcohol  □ Marijuana  □ Nicotine  □ Inhalant  □ Opiates (Vicodin, etc.)  □ Non-prescribed stimulant (Adderall, etc.)  □ Mushrooms  □ Other substance |
| 1. What substance did you talk to your child about? | Open response |
| 1. When my child and I spoke about their substance use yesterday, I felt UNDERSTOOD | Not at all (0) – Very Much (5) |
| 1. When my child and I spoke about their substance use yesterday, I felt COMFORTABLE | Not at all (0) – Very Much (5) |
| 1. When my child and I spoke about their substance use yesterday, we EASILY DISCUSSED OUR OPINIONS | Not at all (0) – Very Much (5) |
| **NOTE**: All participants continue with Q19. | |
| 1. Yesterday, I had someone who understood my problems | Not at all (0) – Very Much (5) |
| 1. Yesterday, I felt there were people I could talk to if I was upset | Not at all (0) – Very Much (5) |
| 1. Yesterday, my child felt ANXIOUS | Not at all (0) to Very Much (7) |
| 1. Yesterday, my child felt DEPRESSED | Not at all (0) to Very Much (7) |
| 1. Yesterday, my child felt THEIR MOOD WAS UP AND DOWN |  |
| 1. Yesterday, my child felt WORRIED ABOUT BEING ABANDONED | Not at all (0) to Very Much (7) |
| 1. Yesterday, my child felt LITTLE INTEREST IN DOING MUCH OF ANYTHING | Not at all (0) to Very Much (7) |
| 1. Yesterday, my child felt LIKE THEY WANTED TO HURT SOMEONE | Not at all (0) to Very Much (7) |
| 1. Yesterday, my child felt LIKE THEY DIDN’T WANT TO BE AROUND OTHER PEOPLE | Not at all (0) to Very Much (7) |
| 1. Yesterday, my child felt LIKE THEIR RELATIONSHIPS ARE EMPTY | Not at all (0) to Very Much (7) |
| 1. Yesterday, my child acted ON THEIR EMOTIONS | Not at all (0) to Very Much (7) |
| 1. Yesterday, my child acted IMPULSIVELY | Not at all (0) to Very Much (7) |
| 1. Yesterday, my child acted ON IMPULSE WHILE FEELING UPSET | Not at all (0) to Very Much (7) |
| 1. Yesterday, my child acted AGGRESSIVELY TOWARD SOMEONE | Not at all (0) to Very Much (7) |
| 1. Yesterday, my child acted IRRESPONSIBLY | Not at all (0) to Very Much (7) |
| 1. Yesterday, my child SAID SOMETHING OFFENSIVE TO SOMEONE | Not at all (0) to Very Much (7) |
| 1. Yesterday, my child DID SOMETHING DANGEROUS TO SOMEONE | Not at all (0) to Very Much (7) |
| 1. Yesterday, my child LOST THEIR TEMPER | Not at all (0) to Very Much (7) |

**Shortest Possible Length**: 27 items

**Longest Possible Length**: 33 items

**Signal Contingent (FED) Report Instructions, Timing Parameters, and General Functions**

1. Signal contingent report (i.e., random prompts (RPs) triggers are “one time”, they will go off every day for 21 days (3 weeks) during the 3 EMA burst periods.
2. RPs are delivered at a randomly selected time point in each 2-hour 20-minute time block during weekdays and 2-hour 45-minute time block during weekends.
3. RPs are delivered between the hours of 3:00 PM to 10: 00 PM at a frequency of 3 times per day during weekdays. During weekends, RPs are delivered between the hours of 11:00 AM to 10:00 PM at a frequency of 4 times per day during weekends.
4. Trigger will begin on the day participants enrolls. Once enrolled, the trigger will start after 0 days.
5. Participants receive a push notification prompt followed by a text message that will remind the participant to complete RPs. If the participant fails to respond to the prompt for the RP within 10 minutes of the trigger, then another push notification prompt followed by a text message will be delivered.
6. If the RP is not completed, the report will disappear after 30 minutes, and the participant will not have the opportunity to fill it out until the next randomly selected time point in the next time block.

# Random Prompt - Adolescent

| **ITEM** | **RESPONSE OPTION** |
| --- | --- |
| 1. Check any substances you used since your last report. | □ None  □ Alcohol  □ Marijuana  □ Nicotine  □ Energy Drink (Red bull, etc.)  □ Inhalant  □ Opiates (Vicodin, etc.)  □ Non-prescribed stimulant (Adderall, etc.)  □ Stimulant as prescribed (Adderall, etc.)  □ Mushrooms  □ Other |
| **NOTE**: Q2-Q3 will be administered for each substance endorsed in Q1. | |
| 1. Enter the time you began using [pipe name of substance from Question 1] | (Hour and minute response option) |
| 1. Enter the time you finished using [pipe name of substance from Question 1] | (Hour and minute response option) |
| **NOTE**: Q4-6 will be administered if a participant endorses alcohol use in Q1. | |
| 1. Check the box beside all the types of alcohol you drank? | □ Beer (Bud Light, etc.)  □ Malt Liquor (Colt 45, etc.)  □ Liquor (straight or mixed)  □ Wine (Merlot, etc.)  □ Wine Cooler or hard seltzer (Boone’s, etc.)  □ Fortified Wine (Mad Dog 20/20, etc.)  □ I did not drink yesterday |
| 1. Enter the number of standard drinks or ounces of [pipe response from Q4] you drank since your last report. |  |
| 1. You entered [show amount recorded] standard drinks or [show amount] ounces of alcohol. Is this correct? | □ Yes  □ No |
| **NOTE:** Q7 will be administered if a participant endorses cannabis use in Q1. | |
| 1. How high did you feel since your last report? | 0 (not at all) to 100 (highest I have ever felt) |
| **NOTE:** All participants continue with Q8. | |
| 1. Where are you? | □ Home  □ Friend’s house  □ School  □ Work  □ At a party  □ Restaurant  □ Outside in public place (park, etc.)  □ Inside in public place (store, library, etc.)  □ Car, bus, other transportation  □ Elsewhere |
| 1. Who is with you? | □ No one  □ Friend(s) I drink with  □ Friend(s) I don’t drink with  □ Romantic partner I drink with  □ Romantic partner I don’t drink with  □ Child/children  □ Mother  □ Father  □ Brother(s) or sister(s)  □ Other relative(s)  □ Friend’s parents  □ Coworkers  □ Other(s) |
| 1. Since your last report, did you have or could get any of the following substances? (select all that apply) | □ None  □ Alcohol  □ Marijuana  □ Nicotine  □ Energy Drink (Red bull, etc.)  □ Inhalant  □ Opiates (Vicodin, etc.)  □ Non-prescribed stimulant (Adderall, etc.)  □ Stimulant as prescribed (Adderall, etc.)  □ Mushrooms  □ Other (please specify) |
| 1. How strong is your urge to drink alcohol right now? | No urge (0) – Strongest ever (10) |
| 1. How strong is your urge to use marijuana right now? | No urge (0) – Strongest ever (10) |
| 1. How strong is your urge to use nicotine right now? | No urge (0) – Strongest ever (10) |
| 1. Have you seen or talked to one of your parents/caregivers since the last report? | □ Yes, in person  □ Yes, by phone  □ Yes, by text  □ Yes, by instant messaging or social media  □ Yes, by video chat  □ No, I did not talk with my parent or caregiver |
| **NOTE**: Q16-24 will only be administered if participant answers “yes” (i.e., they were in contact with a caregiver since the last report) to Q14. | |
| 1. When you spoke to your parent or caregiver by [pipe Question 8 response], how did your parent or caregiver behave toward you? | -50 = Distant/Cold to 50 = Warm/Friendly |
| 1. When you spoke to your parent or caregiver by [pipe Question 8 response], how did your parent or caregiver behavior toward you? | -50 = Shy/Passive to 50 = Confident/Pushy |
| 1. When you spoke to your parent or caregiver by [pipe Question 8 response], how did you behave toward your them? | -50 = Distant/Cold to 50 = Warm/Friendly |
| 1. When you spoke to your parent or caregiver by [pipe Question 8 response], how did you behave toward your them? | -50 = Shy/Passive to 50 = Confident/Pushy |
| 1. Since your last report, your parent or caregiver PRAISED you (e.g., told you that you did a good job). | 0 (strongly disagree) to 100 (strongly agree) |
| 1. Since your last report, your parent or caregiver SUPPORTED you. | 0 (strongly disagree) to 100 (strongly agree) |
| 1. Since your last report, your parent or caregiver SHOWED YOU LOVE. | 0 (strongly disagree) to 100 (strongly agree) |
| 1. Since your last report, your parent or caregiver CRITICIZED you. | 0 (strongly disagree) to 100 (strongly agree) |
| 1. Since your last report, your parent or caregiver was ANNOYED by you. | 0 (strongly disagree) to 100 (strongly agree) |
| 1. Since your last report, you and your parent or caregiver DISAGREED. | 0 (strongly disagree) to 100 (strongly agree) |
| **NOTE**: The following supplemental items (S1-S4) will only be administered if a participant answers “no, I did not talk with parent or caregiver” to Q14. | |
| S1. Right now, I could resist my friend’s offer of a drink or another drug. | 0 (not at all) to 100 (extremely) |
| S2. Right now, it would be pretty easy for my friend to change my mind. | 0 (not at all) to 100 (extremely) |
| S3. Right now, I would do something I knew was wrong to stay on my friend’s good side. | 0 (not at all) to 100 (extremely) |
| S4. Right now, I would say something I don’t believe so my friends respect me more. | 0 (not at all) to 100 (extremely) |
| **NOTE**: All participants continue with Q25. | |
| 1. How motivated are you to cut down or stop drinking alcohol right now? | Not Motivated (1) – Extremely Motivated (10) |
| 1. My parent or caregiver knows where I am right now. | 0 (strongly disagree) to 100 (strongly agree) |
| 1. Since your last report, which of the following areas did you and your parent or caregiver have a disagreement or conflict about? (check all that apply) | □ We didn’t have a conflict/disagreement  □ Chores/Responsibilities  □ Dressing – clothes, hair  □ School – attendance, grades, homework  □ Friends  □ Disclosure/Secrecy  □ Money  □ Electronics Use  □ Social Media  □ Substance Use |
| **NOTE**: Q28-Q29 will only be administered if a participant chooses a response indicating a disagreement or conflict on Q27. | |
| 1. How did your parent or caregiver try to resolve this disagreement or conflict? (check all that apply) | □ Threatened a punishment/consequence  □ Gave a punishment/consequence  □ Tried to discuss issue calmly  □ Did discuss issue calmly  □ Got information to back up their side  □ Brought in someone to help settle things  □ Left the room to cool down  □ Yelled, insulted or swore  □ Refused to talk about it  □ Cried  □ Threw, smashed, hit, or kicked something  □ None of the above |
| 1. How did you try to resolve this disagreement or conflict? (check all that apply) | □ Tried, but failed, to discuss the issue calmly  □ Did discuss the issue calmly  □ Got information to back up my side  □ Brought in someone to help settle things  □ Left the room to cool down  □ Yelled, insulted or swore  □ Refused to talk about it  □ Cried  □ Threw, smashed, hit, or kicked something  □ None of the above |
| **NOTE**: If a participant selects “we didn’t have a conflict/disagreement” on Question 27, they will receive the following three questions. | |
| S7. I want to stop what I’m doing so I can feel better | 1 (strongly disagree) to 7 (strongly agree) |
| S8. Right now, my emotions are getting in my way. | 1 (strongly disagree) to 7 (strongly agree) |
| S9. I can keep doing what I’m doing right now, regardless of how I feel. | 1 (strongly disagree) to 7 (strongly agree) |
| **NOTE**: Q30-39 will not be administered if a participant reports that they did not talk to their caregiver since the last report (“No, I did not talk with my parent or caregiver” to Q14). | |
| 1. Since your last report, did your parent or caregiver talk to you about (check all that apply) | □ Alcohol  □ Marijuana  □ Nicotine  □ Inhalant  □ Opiates (Vicodin, etc.)  □ Non-prescribed stimulant (Adderall, etc.)  □ Mushrooms  □ Other substance  □ I did not talk to my parent or caregiver about substance use |
| 1. What substance did your parent or caregiver talk to you about? | Open response |
| **Note**: Q31 will only be administered if an adolescent selects “another substance” for Q30. If an adolescent endorses talking to their caregiver about substance use in Q30, they will be asked about the corresponding substance in Q32-34. For example, if they report talking to their caregiver about alcohol, they will receive Q32. If they report talking to their caregiver about nicotine, they will receive Q34. Q36-Q38 will be administered whenever an adolescent endorses talking with their caregiver about substance use in Q30. | |
| 1. Since your last report, did your parent or caregiver talk to you about (check all that apply) | □ Not drinking alcohol  □ How to resist pressure to drink alcohol  □ Rules about drinking alcohol  □ Discipline about alcohol  □ Encouraged you not to drink alcohol  □ Encouraged you to use a skill to not drink  □ None of the above |
| 1. Since your last report, did you parent or caregiver talk to you about (check all that apply) | □ Not using marijuana  □ How to resist pressure to use marijuana  □ Rules about using marijuana  □ Discipline about marijuana  □ Encouraged you not to use marijuana  □ Encouraged you to use a skill to not use marijuana  □ None of the above |
| 1. Since your last report, did you parent or caregiver talk to you about (check all that apply) | □ Not using nicotine  □ How to resist pressure to use nicotine  □ Rules about using nicotine  □ Discipline about nicotine  □ Encouraged you not to use nicotine  □ Encouraged you to use a skill to not use nicotine  □ None of the above |
| 1. Since your last report, did you parent or caregiver talk to you about (check all that apply) | □ Not using [pipe response to Question 22]  □ How to resist pressure to use [pipe response to Question 22]  □ Rules about using [pipe response to Question 22]  □ Discipline about [pipe response to Question 22]  □ Encouraged you not to use [pipe response to Question 22]  □ Encouraged you to use a skill to not use [pipe response to Question 22]  □ None of the above |
| 1. Since your last report, when my parent or caregiver and I spoke about my substance use, I felt UNDERSTOOD. | Not at all (0) – Very Much (5) |
| 1. Since your last report, when my parent or caregiver and I spoke about my substance use, I felt COMFORTABLE. | Not at all (0) – Very Much (5) |
| 1. Since your last report, when my caregiver and I spoke about my substance use, we EASILY DISCUSSED OUR OPINIONS | Not at all (0) – Very Much (5) |
| **NOTE**: All participants continue with Q39. | |
| 1. How LOVED BY YOUR PARENT OR CAREGIVER do you feel right now? | Not at all (0) – Extremely (100) |
| 1. How ANGRY do you feel right now? | Not at all (0) – Extremely (100) |
| 1. How NERVOUS do you feel right now? | Not at all (0) – Extremely (100) |
| 1. How SAD do you feel right now? | Not at all (0) – Extremely (100) |
| 1. How HAPPY do you feel right now? | Not at all (0) – Extremely (100) |
| 1. How EXCITED do you feel right now? | Not at all (0) – Extremely (100) |
| 1. How RELAXED do you feel right now? | Not at all (0) – Extremely (100) |

**Shortest Possible Length**: 25 items

**Longest Possible Length**: 46 items

**Signal Contingent (FED) Report Instructions, Timing Parameters, and General Functions**

1. Signal contingent report (i.e., random prompts (RPs) triggers are “one time”, they will go off every day for 21 days (3 weeks) during the 3 EMA burst periods.
2. RPs are delivered at a randomly selected time point in each 2-hour 20 minute time block during weekdays and 2-hour 45 minute time block during weekends.
3. RPs are delivered between the hours of 3:00 PM to 10: 00 PM at a frequency of 3 times per day during weekdays. During weekends, RPs are delivered between the hours of 11:00 AM to 10:00 PM at a frequency of 4 times per day during weekends.
4. Trigger will begin on the day participants enrolls. Once enrolled, the trigger will start after 0 days.
5. Participants receive a push notification prompt followed by a text message that will remind the participant to complete RPs. If the participant fails to respond to the prompt for the RP within 10 minutes of the trigger, then another push notification prompt followed by a text message will be delivered.
6. If the RP is not completed, the report will disappear after 30 minutes, and the participant will not have the opportunity to fill it out until the next randomly selected time point in the next time block.

# Random Prompt – Caregiver

| **ITEM** | **RESPONSE OPTION** |
| --- | --- |
| 1. Check any substances your child used since your last report. | □ None  □ Alcohol  □ Marijuana  □ Nicotine  □ Energy Drink (Red bull, etc.)  □ Inhalant  □ Opiates (Vicodin, etc.)  □ Non-prescribed stimulant (Adderall, etc.)  □ Stimulant as prescribed (Adderall, etc.)  □ Mushrooms  □ Other |
| 1. Check any substances you used since your last report. | □ None  □ Alcohol  □ Marijuana  □ Nicotine  □ Energy Drink (Red bull, etc.)  □ Inhalant  □ Opiates (Vicodin, etc.)  □ Non-prescribed stimulant (Adderall, etc.)  □ Stimulant as prescribed (Adderall, etc.)  □ Mushrooms  □ Other |
| **NOTE:** Q3 and Q4 will only be administered if a participant reports substance use since the last report. | |
| 1. Enter the time you began using [pipe name of substance from Question 2] | (Hour and minute response option) |
| 1. Enter the time you finished using [pipe name of substance from Question 1] | (Hour and minute response option) |
| **NOTE:** All participants continue with Q5. | |
| 1. Where are you? | □ Home  □ Friend’s house  □ School  □ Work  □ At a party  □ Restaurant  □ Outside in public place (park, etc.)  □ Inside in public place (store, library, etc.)  □ Car, bus, other transportation  □ Elsewhere |
| 1. Who is with you? | □ No one  □ Child participating in this study  □ My other children  □ Significant Other  □ Friend(s) I drink with  □ Friend(s) I don’t drink with  □ Friend of child participating in this study  □ Mother  □ Father  □ Brother(s) or sister(s)  □ Other relative(s)  □ Coworkers  □ Other(s) |
| 1. Have you seen or talked to your child since the last report? | □ Yes, in person  □ Yes, by phone  □ Yes, by text  □ Yes, by instant messaging or social media  □ Yes, by video chat  □ No, I did not talk with my child |
| **NOTE**: Q8-Q17 will only be administered if participant answers “yes” (i.e., they were in contact with their child since the last report) to Q7. | |
| 1. When you spoke to your child by [pipe Question 7 answer], how did your child behave toward you? | -50 = Distant/Cold to 50 = Warm/Friendly |
| 1. When you spoke to your child by [pipe Question 7 answer], how did your child behavior toward you during this interaction? | -50 = Shy/Passive to 50 = Confident/Pushy |
| 1. When you spoke to your child by [pipe Question 7 answer], how did you behave toward them during this interaction? | -50 = Distant/Cold to 50 = Warm/Friendly |
| 1. When you spoke to your child by [pipe Question 7 answer], how did you behave toward them child during this interaction? | -50 = Shy/Passive to 50 = Confident/Pushy |
| 1. Since your last report, you PRAISED your child (e.g., told them they did a good job). | 0 (strongly disagree) to 100 (strongly agree) |
| 1. Since your last report, you SUPPORTED your child. | 0 (strongly disagree) to 100 (strongly agree) |
| 1. Since your last report, you SHOWED LOVE to your child. | 0 (strongly disagree) to 100 (strongly agree) |
| 1. Since your last report, you CRITICIZED your child. | 0 (strongly disagree) to 100 (strongly agree) |
| 1. Since your last report, you WERE ANNOYED by your child. | 0 (strongly disagree) to 100 (strongly agree) |
| 1. Since your last report, you DISAGREED with your child. | 0 (strongly disagree) to 100 (strongly agree) |
| **NOTE**: The following supplemental items (S1-S8) will only be administered if a participant answers “no, I did not talk with my child” to Q7. | |
| S1. How stressed are you feeling right now? | 0 (not at all) to 100 (extremely) |
| S2. How certain do you feel that you can deal with all the things that you have to do right now? | 0 (not at all) to 100 (extremely) |
| S3. How confident do you feel about your ability to handle all the demands on you right now? | 0 (not at all) to 100 (extremely) |
| S4. Since your last report, which of these things caused you stress? (check all that apply) | □ Work at home  □ Work at a job  □ Demands made by your family  □ Tension with a coworker  □ Tension with a spouse  □ Tension with your children  □ Something else  □ Nothing caused me stress |
| **NOTE**: All participants will continue with Q18. | |
| 1. I know what my child is doing right now. | 0 (strongly disagree) to 100 (strongly agree) |
| 1. I know who my child is with right now. | 0 (strongly disagree) to 100 (strongly agree) |
| 1. I know where my child is right now. | 0 (strongly disagree) to 100 (strongly agree) |
| 1. I know whether my child has access to alcohol or other drugs right now. | 0 (strongly disagree) to 100 (strongly agree) |
| 1. Where is your child? | □ Home  □ Friend’s house  □ School  □ Work  □ At a party  □ Restaurant  □ Outside in public place (park, etc.)  □ Inside in public place (store, library, etc.)  □ Car, bus, other transportation  □ Elsewhere  □ I don’t know |
| 1. Since your last report, which of the following areas did you and your child have a disagreement or conflict about? (check all that apply) | □ We didn’t have a conflict/disagreement  □ Chores/Responsibilities  □ Dressing – clothes, hair  □ School – attendance, grades, homework  □ Friends  □ Disclosure/Secrecy  □ Money  □ Electronics Use  □ Social Media  □ Substance Use |
| **NOTE**: Q24-25 will only be administered if a participant chooses a response indicating a disagreement or conflict on Q23. | |
| 1. How did you try to resolve this disagreement or conflict? (check all that apply) | □ Threatened to give a punishment or consequence  □ Gave a punishment or consequence  □ Tried to discuss the issue calmly  □ Did discuss the issue calmly  □ Got information to back up my side  □ Brought in someone to help settle things  □ Left the room to cool down  □ Yelled, insulted or swore  □ Refused to talk about it  □ Cried  □ Threw, smashed, hit, or kicked something  □ None of the above |
| 1. How did your child try to resolve this disagreement or conflict? (check all that apply) | □ Tried, but failed, to discuss the issue calmly  □ Did discuss the issue calmly  □ Got information to back up their side  □ Brought in someone to help settle things  □ Left the room to cool down  □ Yelled, insulted or swore  □ Refused to talk about it  □ Cried  □ Threw, smashed, hit, or kicked something  □ None of the above |
| **NOTE**: If a participant selects “we didn’t have a conflict/disagreement” on Q23, they will receive the following three questions. | |
| S9. I want to stop what I’m doing so I can feel better | 1 (strongly disagree) to 7 (strongly agree) |
| S10. Right now, my emotions are getting in my way. | 1 (strongly disagree) to 7 (strongly agree) |
| S11. I can keep doing what I’m doing right now, regardless of how I feel. | 1 (strongly disagree) to 7 (strongly agree) |
| **NOTE**: Q26-Q34 will not be administered if a caregiver reports that they did not talk to their child since the last report (“No, I did not talk with my child” to Q7). | |
| 1. Since your last report, did your parent or caregiver talk to you about (check all that apply) | □ Alcohol  □ Marijuana  □ Nicotine  □ Inhalant  □ Opiates (Vicodin, etc.)  □ Non-prescribed stimulant (Adderall, etc.)  □ Mushrooms  □ Other substance  □ I did not talk to my parent or caregiver about substance use |
| 1. What substance did your parent or caregiver talk to you about? | Open response |
| **Note**: Q27 will only be administered if a caregiver selects “another substance” for Q26. If a caregiver endorses talking to their child about substance use in Q26, they will be asked about the corresponding substance in Q28-31. For example, if they report talking to their child about alcohol, they will receive Q28. If they report talking to their child about nicotine they will receive Q30. Q32-34 will be administered whenever a caregiver endorses talking with their child about substance use in Q26. | |
| 1. Since your last report, did you talk to your child about (check all that apply) | □ Not drinking alcohol  □ How to resist pressure to drink alcohol  □ Rules about drinking alcohol  □ Discipline about alcohol  □ Encouraged your child not to drink alcohol  □ Encouraged your child to use a skill to not drink  □ None of the above |
| 1. Since your last report, did you talk to your child about (check all that apply) | □ Not using marijuana  □ How to resist pressure to use marijuana  □ Rules about using marijuana  □ Discipline about marijuana  □ Encouraged your child not to use marijuana  □ Encouraged your child to use a skill to not use marijuana  □ None of the above |
| 1. Since your last report, did you talk to your child about (check all that apply) | □ Not using nicotine  □ How to resist pressure to use nicotine  □ Rules about using nicotine  □ Discipline about nicotine  □ Encouraged your child not to use nicotine  □ Encouraged your child to use a skill to not use nicotine  □ None of the above |
| 1. Since your last report, did you talk to your child about (check all that apply) | □ Not using [pipe response to Question 22]  □ How to resist pressure to use [pipe response to Question 22]  □ Rules about using [pipe response to Question 22]  □ Discipline about [pipe response to Question 22]  □ Encouraged your child not to use [pipe response to Question 22]  □ Encouraged your child to use a skill to not use [pipe response to Question 22]  □ None of the above |
| 1. Since your last report, when my child and I spoke about their substance use, I felt UNDERSTOOD | Not at all (0) – Very Much (5) |
| 1. Since your last report, when my child and I spoke about their substance use, I felt COMFORTABLE | Not at all (0) – Very Much (5) |
| 1. Since your last report, when my child and I spoke about their substance use, we EASILY DISCUSSED OUR OPINIONS | Not at all (0) – Very Much (5) |
| **NOTE**: All participants continue with Q35. | |
| 1. How LOVED by your child do you feel right now? | Not at all (0) – Extremely (100) |
| 1. How ANGRY do you feel right now? | Not at all (0) – Extremely (100) |
| 1. How NERVOUS do you feel right now? | Not at all (0) – Extremely (100) |
| 1. How SAD do you feel right now? | Not at all (0) – Extremely (100) |
| 1. How HAPPY do you feel right now? | Not at all (0) – Extremely (100) |
| 1. How EXCITED do you feel right now? | Not at all (0) – Extremely (100) |
| 1. How RELAXED do you feel right now? | Not at all (0) – Extremely (100) |

**Shortest Possible Length**: 25 items

**Longest Possible Length**: 42 items

**Mental Health Update Report Instructions, Timing Parameters, and General Functions**

**Event-Contingent Report**

1. The mental health update will be added to participant morning reports each Saturday.
2. Considering the mental health update will be added to morning reports, the same reminders for morning reports will be used for the mental health update reports.
3. If a participant does not complete the mental health update report on Saturday, it will be added to their morning report on Sunday.

# Mental Health Update Report - Adolescent (therapy session)

| **ITEM** | **RESPONSE OPTION** |
| --- | --- |
| 1. Did you have a therapy session this past week? | □ Yes  □ No, I did not have therapy this week  □ No, I missed my appointment  □ No, I quit therapy early  □ No, I finished therapy |
| **NOTE**: Participants who endorse “Yes” to Q1 will be asked Q2. Any “No” response will be directed to additional surveys listed in the subsequent sections. | |
| 1. How many therapy sessions did you have this past week? | 1-7 |
| 1. Even if it was just for a few minutes, did you parent or caregiver join you in therapy this past week? | □ Yes  □ No |
| **NOTE:** If Q3 is answered “Yes,” participants will be administered Q4. | |
| 1. How long was your parent or caregiver in the therapy session for? | (Hour and minute response option) |
| **NOTE:** All participants will continue with Q5. | |
| 1. How long was your therapy session? | (Hour and minute response option) |
| 1. How did YOUR THERAPIST BEHAVE toward you during your therapy session this past week? | -50 (Distant/Cold) to 50 (Warm/Friendly) |
| 1. How did YOUR THERAPIST BEHAVE toward you during your therapy session this past week? | -50 (Shy/Passive) to 50 (Confident/Pushy) |
| 1. How did YOU BEHAVE toward your therapist during your therapy session this past week? | -50 (Distant/Cold) to 50 (Warm/Friendly) |
| 1. How did YOU BEHAVE toward your therapist during your therapy session this past week? | -50 (Shy/Passive) to 50 (Confident/Pushy) |
| **Note**: Instructions for Q10-Q15. Please answer the following questions based on your relationship with your therapist over the past week. | |
| 1. My therapist and I worked towards goals we both agree on. | 0 (not at all) 1 (a little) 2 (moderately) 3 (quite a bit), 4 (very much), 5 (completely) |
| 1. My therapist and I agreed on what is important for me to work on. | 0 (not at all) 1 (a little) 2 (moderately) 3 (quite a bit), 4 (very much), 5 (completely) |
| 1. My therapist and I respected each other. | 0 (not at all) 1 (a little) 2 (moderately) 3 (quite a bit), 4 (very much), 5 (completely) |
| 1. I believe my therapist liked me. | 0 (not at all) 1 (a little) 2 (moderately) 3 (quite a bit), 4 (very much), 5 (completely) |
| 1. I believe the things I do in therapy will help me to accomplish the changes that I want. | 0 (not at all) 1 (a little) 2 (moderately) 3 (quite a bit), 4 (very much), 5 (completely) |
| 1. I felt that my therapist would care about me even if I did things they do not approve of. | 0 (not at all) 1 (a little) 2 (moderately) 3 (quite a bit), 4 (very much), 5 (completely) |
| 1. How helpful was therapy this past week? | 0 (not at all helpful) – 10 (extremely helpful) |
| 1. How much did you participate in your therapy this past week? | Not At All (1) – Extremely (10) |
| 1. How important was therapy to you this past week? | Not important (1) – Extremely Important (10) |
| 1. How important was your therapy to your parent or caregiver this past week? | Not important (1) – Extremely Important (10) |
| 1. How motivated were you to participate in treatment this past week? | Not Motivated (1) – Extremely Motivated (10) |
| 1. What did you talk about in therapy this week? (select all that apply) | □ Emotions, like anger, anxiety, or depression  □ Thoughts, like unhelpful thinking patterns  □ Stressors or traumas  □ Substance use or cravings  □ Friends, peers, or romantic partner  □ Parent or Caregiver  □ Other family members  □ School  □ Work  □ Activities/Hobbies  □ None of the above  □ Other (please specify) |
| **NOTE**: Q22 will be administered if a participant endorses other in Q21. | |
| 1. What else did you talk about in therapy this week? | Open response |
| 1. What did you learn or practice in therapy this week? (select all that apply) | □ why I’m in therapy  □ better ways to talk with my caregiver(s) or parent(s)  □ better ways of talking with my other family members  □ better ways of talking with my friends or romantic partner  □ ways to relax when I have strong feelings  □ ways to deal with my substance use  □ an activity to help me face my fears like trauma, social anxiety, school anxiety  □ how to better solve problems  □ None of the above |
| 1. During therapy this week, did you learn or practice how… (select all that apply) | □ fun activities can improve my mood  □ thoughts can change feelings and behaviors  □ to spot unhelpful thoughts or try more helpful ways of thinking  □ what happens before or after a behavior, like an argument or substance use, can help change my future behavior  □ rewards and consequences can impact my behavior  □ to do anything else not included in this list  □ None of the above |
| **NOTE**: If a participant selects “to do anything else not included in this list” they will answer Q25. | |
| 1. What did you learn or practice? | Open response |
| **NOTE**: For each answer selected in Q23 and 24, participants will be asked Q26-28. | |
| 1. How helpful was learning or practicing [enter skill selected from above]? | 0 (not at all helpful) – 10 (extremely helpful) |
| 1. During your therapy session, how much time did you spend learning or practicing [enter skill selected from above]? | (Hour and minute response option) |
| 1. [enter skill selected from above]: In which areas did you and your therapist talk about using this? (select all that apply) | □ Emotions, like anger, anxiety, or depression  □ Thoughts, like unhelpful thinking patterns  □ Stressors or traumas  □ Substance use or cravings  □ Friends, peers, or romantic partner  □ Parent or Caregiver  □ Other family members  □ School  □ Work  □ Activities/Hobbies  □ None of the above  □ Other (please specify) |
| **NOTE**: All participants will continue at Q29. | |
| 1. This past week, how much did your parent or caregiver SUPPORT YOUR MENTAL HEALTH? | Not at all (0) to Very Much (10) |
| 1. This past week, how much did your parent or caregiver SUPPORT YOUR SUBSTANCE USE GOALS? | Not at all (0) to Very Much (10) |
| 1. This past week, how much did your parent or caregiver HELP YOU PRACTICE WAYS OF MANAGING YOUR MENTAL HEALTH OR SUBSTANCE USE? | Not at all (0) to Very Much (10) |
| 1. This past week, how much did your parent or caregiver ENCOURAGE YOU TO ATTEND THERAPY? | Not at all (0) to Very Much (10) |
| 1. This past week, how much did you ENGAGE IN FAMILY ACTIVITIES? | Not at all (0) to Very Much (10) |
| 1. This past week, how many days did you have meals with your family. | 0 days – 7 days |
| 1. How much do you think therapy so far has helped you with your SUBSTANCE USE? | 0 (not at all) to 10 (completely or extremely) |
| 1. How much do you think therapy so far has helped you with your MENTAL HEALTH? | 0 (not at all) to 10 (completely or extremely) |
| 1. How much do you think therapy so far has helped your RELATIONSHIP WITH YOUR PARENT(S) OR CAREGIVER(S)? | 0 (not at all) to 10 (completely or extremely) |
| 1. How much do you think therapy so far has helped your RELATIONSHIPS WITH YOUR FRIENDS? | 0 (not at all) to 10 (completely or extremely) |
| 1. How much do you think therapy so far has helped you with SCHOOL, EDUCATION, OR WORK ? | 0 (not at all) to 10 (completely or extremely) |
| 1. Check all the recovery supports you attended or used this past week? ) | □ Smart phone app – mental health  □ Smart phone app – substance use  □ Peer recovery coach  □ AA/NA/MA meeting  □ SMART meeting  □ Group therapy  □ Faith-based support  □ Other recovery support  □ I did not use another recovery support |
| **NOTE**: If a participant endorses any answer other than “I did not use another recovery support” they will continue with Q42-43. They will be asked these questions for each recovery support they endorse in Question 40. Participants will also complete Q41 if they endorse “Other recovery support.” Participants who endorse “I did not use another recovery support” will continue with Q44. | |
| 1. What was the recovery support you used? | (open text) |
| 1. How many days did you use this support in the past week? | 1 day – 7 days |
| 1. How helpful was [pipe Q40 response]? | 0 (not at all helpful) – 10 (extremely helpful) |
| 1. This past week, did you take any prescribed medication for your mental health or substance use? (select all that apply) | □ Yes, for my mental health  □ Yes, for my substance use  □ No  □ Not sure |
| **NOTE**: If a participant endorses “Yes” or “Not sure” to Q44 they will answer Q45-47, if they answer “No” they will be directed to Q48. | |
| 1. This past week, how many days did you forget to take your medication? | 0 days – 7 days |
| 1. This past week, how much did your parent or caregiver help you with taking your medication? | Not at all (0) to Very Much (10) |
| 1. This past week, how helpful was this medication? | 0 (not at all helpful) – 10 (extremely helpful) |
| **NOTE**: All participants continue with Q48. | |
| 1. Did you experience any of the following events during the past week. (select all that apply) | □ I was targeted or harassed  □ I saw or heard negative, hurtful, or offensive messages or stereotypes  □ I was ignored, isolated, or made to feel invisible  □ I was misunderstood  □ People stared at me  □ I was not accepted  □ Someone made me feel uncomfortable or unsafe  □ Someone made me feel less of a human  □ None of the above. |
| **NOTE**: Q49 will be administered for each response other than “None of the above” to Question 48. | |
| 1. What do you think is the reason(s) for this experience? (select all that apply) | □ My sexual orientation  □ My gender identity  □ My gender expression  □ My race/ethnicity  □ My substance Use  □ My mental health  □ My body size  □ My physical Disability  □ My social class  □ Other (please specify)  □ None of the above |

**Shortest Possible Length**: 36 items

**Longest Possible Length**: 49 items

**Mental Health Update Report Instructions, Timing Parameters, and General Functions**

1. The mental health update will be added to participant morning reports each Saturday.
2. Considering the mental health update will be added to morning reports, the same reminders for morning reports will be used for the mental health update reports.
3. If a participant does not complete the mental health update report on Saturday, it will be added to their morning report on Sunday.

# Mental Health Update Report - Adolescent (Did Not Have Therapy/Missed Session)

| **ITEM** | **RESPONSE OPTION** |
| --- | --- |
| **NOTE**: Q1 will be administered if a participant selects “No, I did not have therapy this week.” Q2 will be administered if a participant selects “No, I missed my appointment.” | |
| 1. What were the reason(s) for not having a therapy session this past week? (select all that apply) | □ I was too busy or had a conflict in my schedule  □ My therapist was no available  □ I had transportation issues  □ I could not afford transportation  □ I could not afford the therapy session  □ I was physically sick  □ My mental health symptoms  □ I did not want to talk to my therapist  □ I do not find therapy helpful  □ A conflict with my caregiver(s)  □ I had family or personal problems  □ Other (please specify) |
| 1. What were the reason(s) for missing your therapy session this past week? (select all that apply) | □ I forgot about the session  □ My caregiver forgot about the session  □ I was too busy or had a conflict in my schedule  □ I had transportation issues  □ I could not afford transportation  □ I could not afford the therapy session  □ I was physically sick  □ My mental health symptoms  □ I did not want to talk to my therapist  □ I do not find therapy helpful  □ A conflict with my caregiver(s)  □ I had family or personal problems  □ Other (please specify) |
| **Note**: Instructions for Q3-Q8. Please answer the following questions based on your relationship with your therapist over the past week, even if you missed a session or did not have one. | |
| 1. My therapist and I worked towards goals we both agree on. | 0 (not at all) 1 (a little) 2 (moderately) 3 (quite a bit), 4 (very much), 5 (completely) |
| 1. My therapist and I agreed on what is important for me to work on. | 0 (not at all) 1 (a little) 2 (moderately) 3 (quite a bit), 4 (very much), 5 (completely) |
| 1. My therapist and I respected each other. | 0 (not at all) 1 (a little) 2 (moderately) 3 (quite a bit), 4 (very much), 5 (completely) |
| 1. I believe my therapist liked me. | 0 (not at all) 1 (a little) 2 (moderately) 3 (quite a bit), 4 (very much), 5 (completely) |
| 1. I believe the things I do in therapy will help me to accomplish the changes that I want. | 0 (not at all) 1 (a little) 2 (moderately) 3 (quite a bit), 4 (very much), 5 (completely) |
| 1. I felt that my therapist would care about me even if I did things they do not approve of. | 0 (not at all) 1 (a little) 2 (moderately) 3 (quite a bit), 4 (very much), 5 (completely) |
| 1. Check all the recovery supports you attended or used this past week? ) | □ Smart phone app – mental health  □ Smart phone app – substance use  □ Peer recovery coach  □ AA/NA/MA meeting  □ SMART meeting  □ Group therapy  □ Faith-based support  □ Other recovery support  □ I did not use another recovery support |
| **NOTE**: Participants will be asked Q10 if they select “Other recovery support” in Q9. For each recovery support selected, participants will be asked Q11-12. Participants who select “I did not use another recovery support” will continue with Q13. | |
| 1. What was the recovery support you used? | (open text) |
| 1. How many days did you use this support in the past week? | 0 days – 7 days |
| 1. How helpful was [pipe Q9 response]? | 0 (not at all helpful) – 10 (extremely helpful) |
| 1. This past week, did you take any prescribed medication for your mental health or substance use? (select all that apply) | □ Yes, for my mental health  □ Yes, for my substance use  □ No  □ Not sure |
| **NOTE**: If a participant endorses “Yes” to Q13 they will answer Q14-16, if they answer “No” they will be directed to Q17. | |
| 1. This past week, how many days did you forget to take your medication? | 0 days – 7 days |
| 1. This past week, how much did your parent or caregiver help you with taking your medication? | Not at all (0) to Very Much (10) |
| 1. This past week, how helpful was this medication? | 0 (not at all helpful) – 10 (extremely helpful) |
| **NOTE**: All participants continue with Q17. | |
| 1. How important was therapy to you this past week? | Not important (1) – Extremely Important (10) |
| 1. How important was your therapy to your parent or caregiver this past week? | Not important (1) – Extremely Important (10) |
| 1. How motivated were you to participate in treatment this past week? | Not Motivated (1) – Extremely Motivated (10) |
| 1. What did you learn or practice from therapy this past week? (select all that apply) | □ why I’m in therapy  □ better ways to talk with my caregiver(s) or parent(s)  □ better ways of talking with my other family members  □ better ways of talking with my friends or romantic partner  □ ways to relax when I have strong feelings  □ ways to deal with my substance use  □ an activity to help me face my fears like trauma, social anxiety, school anxiety  □ how to better solve problems  □ None of the above |
| 1. This past week, did you learn or practice from therapy how… (select all that apply) | □ fun activities can improve my mood  □ thoughts can change feelings and behaviors  □ to spot unhelpful thoughts or try more helpful ways of thinking  □ what happens before or after a behavior, like an argument or substance use, can help change my future behavior  □ rewards and consequences can impact my behavior  □ to do anything else not included in this list  □ None of the above |
| **NOTE**: For each answer selected in the previous question, participants will be asked Q23. If a participant selects “anything else in your therapy session not included in this list” they will answer Q22 and then Q23. | |
| 1. What did you learn or practice? | Open response |
| 1. How helpful was learning or practicing [enter skill selected from above]? | 0 (not at all helpful) – 10 (extremely helpful) |
| **NOTE**: All participants will continue with Q24. | |
| 1. This past week, how much did your parent or caregiver SUPPORT YOUR MENTAL HEALTH? | Not at all (0) to Very Much (10) |
| 1. This past week, how much did your parent or caregiver SUPPORT YOUR SUBSTANCE USE GOALS? | Not at all (0) to Very Much (10) |
| 1. This past week, how much did your parent or caregiver HELP YOU PRACTICE WAYS OF MANAGING YOUR MENTAL HEALTH OR SUBSTANCE USE? | Not at all (0) to Very Much (10) |
| 1. This past week, how much did your parent or caregiver ENCOURAGE YOU TO ATTEND THERAPY? | Not at all (0) to Very Much (10) |
| 1. This past week, how much did you ENGAGE IN FAMILY ACTIVITIES? | Not at all (0) to Very Much (10) |
| 1. This past week, how many days did you have meals with your family. | 0 days – 7 days |
| 1. How much do you think therapy so far has helped you with your SUBSTANCE USE? | 0 (not at all) to 10 (completely or extremely) |
| 1. How much do you think therapy so far has helped you with your MENTAL HEALTH? | 0 (not at all) to 10 (completely or extremely) |
| 1. How much do you think therapy so far has helped your RELATIONSHIP WITH YOUR PARENT(S) OR CAREGIVER(S)? | 0 (not at all) to 10 (completely or extremely) |
| 1. How much do you think therapy so far has helped your RELATIONSHIPS WITH YOUR FRIENDS? | 0 (not at all) to 10 (completely or extremely) |
| 1. How much do you think therapy so far has helped you with SCHOOL, EDUCATION, OR WORK ? | 0 (not at all) to 10 (completely or extremely) |
| **Directions for the following questions**: Think about the activities and events you do and the experiences you have that are **not** dedicated to things like school, sleeping, or other family responsibilities. These activities could include (but are not limited to) sports teams, work, study groups, school clubs, volunteering, watching television or sports, exercising, and meeting with friends for a meal or a party. | |
| 1. Please enter the number of hours you participated in activities other than school, sleeping, or family responsibilities while you were not using alcohol or drugs in the past week. | hours |
| 1. Please rate on average how much you **enjoyed** activities other than school, sleeping, or family responsibilities while you **were not using alcohol or drugs** in the past week. | □ Unpleasant  □ Neither pleasant nor unpleasant  □ Mildly pleasant  □ Moderately pleasant  □ Very pleasant  □ Extremely pleasant |
| 1. Please enter the number of hours you participated in activities other than school, sleeping, or family responsibilities while you were using alcohol or drugs in the past week. | hours |
| 1. Please rate on average how easy it was to find activities other than school, sleeping, or family responsibilities that did not involve using alcohol or drugs in the past week. | □ Nearly impossible to find  □ Difficult to find  □ Somewhat difficult to find  □ Somewhat easy to find  □ Easy to find  □ Very easy to find |
| 1. Did you experience any of the following events during the past week. (select all that apply) | □ I was targeted or harassed  □ I saw or heard negative, hurtful, or offensive messages or stereotypes  □ I was ignored, isolated, or made to feel invisible  □ I was misunderstood  □ People stared at me  □ I was not accepted  □ Someone made me feel uncomfortable or unsafe  □ Someone made me feel less of a human  □ None of the above. |
| **NOTE**: Q40 will be administered for each response other than “None of the above” to Q39. | |
| 1. What do you think is the reason(s) for this experience? (select all that apply) | □ My sexual orientation  □ My gender identity  □ My gender expression  □ My race/ethnicity  □ My substance Use  □ My mental health  □ My body size  □ My physical Disability  □ My social class  □ Other (please specify)  □ None of the above |

**Shortest Possible Length**: 31 items*

**Longest Possible Length**: 40 items*

*All participants will be administered Q1 from the mental health update report therapy session.

**Mental Health Update Report Instructions, Timing Parameters, and General Functions**

1. The mental health update will be added to participant morning reports each Saturday.
2. Considering the mental health update will be added to morning reports, the same reminders for morning reports will be used for the mental health update reports.
3. If a participant does not complete the mental health update report on Saturday, it will be added to their morning report on Sunday.

# Mental Health Update Report – Adolescent (quit or finished therapy)

| **ITEM** | **RESPONSE OPTION** |
| --- | --- |
| **NOTE**: Participants who select “No, I quit therapy early” will answer Q1 and Q2. Participants who select “No, I finished therapy” will continue with Q3. | |
| 1. Why are you not currently in treatment? (select all that apply) | □ I wanted to handle problem on my own  □ I thought the problem would get better  □ My problem(s) went away  □ I did not know who to see  □ I was scared of what others would think of me being in treatment  □ I thought treatment would not work  □ I thought treatment would take too much time  □ My problem(s) did not bother me much  □ I was scared on being involuntarily hospitalized  □ My past experiences in therapy were not helpful  □ I was unhappy with the treatment I was receiving  □ None of the above |
| 1. Are you not currently in treatment for any of the following reasons? (select all that apply) | □ My family does not support me going to therapy  □ Issues in my family are preventing me from going to therapy  □ I could not get an appointment  □ Treatment was too expensive  □ Transportation to treatment was too expensive  □ Treatment was not covered by insurance  □ I had a hard time getting transportation to treatment  □ It was too hard to find a time for treatment each week  □ None of the above |
| 1. Check all the recovery supports you attended or used this past week? ) | □ Smart phone app – mental health  □ Smart phone app – substance use  □ Peer recovery coach  □ AA/NA/MA meeting  □ SMART meeting  □ Group therapy  □ Faith-based support  □ Other recovery support  □ I did not use another recovery support |
| **NOTE**: Participants will be asked Q4 if they select “Other recovery support” in Q3. For each recovery support selected, participants will be asked Q5 and Q6. Participants who select “I did not use another recovery support” will continue with Q7. | |
| 1. What was the recovery support you used? | (open text) |
| 1. How many days did you use this support in the past week? | 1 day – 7 days |
| 1. How helpful was [pipe Q40 response]? | 0 (not at all helpful) – 10 (extremely helpful) |
| 1. This past week, did you take any prescribed medication for your mental health or substance use? (select all that apply) | □ Yes, for my mental health  □ Yes, for my substance use  □ No  □ Not sure |
| **NOTE**: If a participant endorses “Yes” to Q7 they will answer Q8-Q10, if they answer “No” they will be directed to Q11. | |
| 1. This past week, how many days did you forget to take your medication? | 0 days – 7 days |
| 1. This past week, how much did your parent or caregiver help you with taking your medication? | Not at all (0) to Very Much (10) |
| 1. This past week, how helpful was this medication? | 0 (not at all helpful) – 10 (extremely helpful) |
| **NOTE**: All participants will continue with Q11. | |
| 1. What did you learn or practice from therapy this past week? (select all that apply) | □ better ways to talk with my caregiver(s) or parent(s)  □ better ways of talking with my other family members  □ better ways of talking with my friends or romantic partner  □ ways to relax when I have strong feelings  □ ways to deal with my substance use  □ an activity to help me face my fears like trauma, social anxiety, school anxiety  □ how to better solve problems  □ None of the above |
| 1. This past week, did you learn or practice from therapy how… (select all that apply) | □ fun activities can improve my mood  □ thoughts can change feelings and behaviors  □ to spot unhelpful thoughts or try more helpful ways of thinking  □ what happens before or after a behavior, like an argument or substance use, can help change my future behavior  □ rewards and consequences can impact my behavior  □ to do anything else not included in this list  □ None of the above |
| **NOTE**: For each answer selected in the previous Q11 and Q12, participants will be asked Q14-Q15. If a participant selects “anything else not included in this list” they will answer Q13-15: | |
| 1. What did you learn or practice? | Open response |
| 1. How helpful was learning or practicing [enter skill selected from above]? | 0 (not at all helpful) – 10 (extremely helpful) |
| 1. Regarding your substance use, do you consider yourself to be in recovery right now? | □ Yes  □ No |
| **NOTE**: All participants will continue with Q16. | |
| 1. This past week, how much did your parent or caregiver SUPPORT YOUR MENTAL HEALTH? | Not at all (0) to Very Much (10) |
| 1. This past week, how much did your parent or caregiver SUPPORT YOUR SUBSTANCE USE GOALS? | Not at all (0) to Very Much (10) |
| 1. This past week, how much did your parent or caregiver HELP YOU PRACTICE WAYS OF MANAGING YOUR MENTAL HEALTH OR SUBSTANCE USE? | Not at all (0) to Very Much (10) |
| 1. This past week, how much did your parent or caregiver ENCOURAGE YOU TO ATTEND THERAPY? | Not at all (0) to Very Much (10) |
| 1. This past week, how much did you ENGAGE IN FAMILY ACTIVITIES? | Not at all (0) to Very Much (10) |
| 1. This past week, how many days did you have meals with your family. | 0 days – 7 days |
| **Directions for the following questions**: Think about the activities and events you do and the experiences you have that are **not** dedicated to things like school, sleeping, or other family responsibilities. These activities could include (but are not limited to) sports teams, work, study groups, school clubs, volunteering, watching television or sports, exercising, and meeting with friends for a meal or a party. | |
| 1. Please enter the number of hours you participated in activities other than school, sleeping, or family responsibilities while you were not using alcohol or drugs in the past week. | hours |
| 1. Please rate on average how much you **enjoyed** activities other than school, sleeping, or family responsibilities while you **were not using alcohol or drugs** in the past week. | □ Unpleasant  □ Neither pleasant nor unpleasant  □ Mildly pleasant  □ Moderately pleasant  □ Very pleasant  □ Extremely pleasant |
| 1. Please enter the number of hours you participated in activities other than school, sleeping, or family responsibilities while you were using alcohol or drugs in the past week. | hours |
| 1. Please rate on average how easy it was to find activities other than school, sleeping, or family responsibilities that did not involve using alcohol or drugs in the past week. | □ Nearly impossible to find  □ Difficult to find  □ Somewhat difficult to find  □ Somewhat easy to find  □ Easy to find  □ Very easy to find |
| 1. Did you experience any of the following events during the past week. (select all that apply) | □ I was targeted or harassed  □ I saw or heard negative, hurtful, or offensive messages or stereotypes  □ I was ignored, isolated, or made to feel invisible  □ I was misunderstood  □ People stared at me  □ I was not accepted  □ Someone made me feel uncomfortable or unsafe  □ Someone made me feel less of a human  □ None of the above. |
| **NOTE**: Q27 will be administered for each response other than “None of the above” to Question 27. | |
| 1. What do you think is the reason(s) for this experience? (select all that apply) | □ My sexual orientation  □ My gender identity  □ My gender expression  □ My race/ethnicity  □ My substance Use  □ My mental health  □ My body size  □ My physical Disability  □ My social class  □ Other (please specify)  □ None of the above |

**Shortest Possible Length**: 16 items*

**Longest Possible Length**: 28 items*

*All participants will be administered Q1 from the mental health update report therapy session.

**Mental Health Update Report Instructions, Timing Parameters, and General Functions**

1. The mental health update will be added to participant morning reports each Saturday.
2. Considering the mental health update will be added to morning reports, the same reminders for morning reports will be used for the mental health update reports.
3. If a participant does not complete the mental health update report on Saturday, it will be added to their morning report on Sunday.

# Mental Health Update Report – Caregiver (therapy session)

| **ITEM** | **RESPONSE OPTION** |
| --- | --- |
| 1. Did your child have a therapy session this past week? | □ Yes  □ No, they did not have therapy this week  □ No, they missed their appointment  □ No, they quit therapy early  □ No, they finished therapy  □ I am not sure if they had a therapy session |
| **NOTE**: If a caregiver selects “Yes” to Q1, they will proceed with this survey. If a caregiver selects “No, they did not have therapy this week” or “No, they missed their appointment” they will complete the Caregiver (did not have therapy/missed session) version of the MHU. If a caregiver selects “No, they quit therapy early” or “No, they finished therapy,” they will complete the Caregiver (quit or finished therapy) version of the MHU. If a caregiver selects “I am not sure if they had a therapy session,” they will complete the Caregiver (not sure if child had a therapy session) version of the MHU. | |
| 1. How many therapy sessions did your child have this past week? | 1-7 |
| **NOTE:** If Q3 is answered “Yes,” participants will answer Q4 then proceed to Q12. If a caregiver selects “No” to Q3, they will be asked Q5-Q11. | |
| 1. Even if it was just for a few minutes, did you join your child’s therapy this past week? | □ Yes  □ No |
| 1. How long were you in the therapy session for? | (Hour and minute response option) |
| 1. Did any of the following factors make it difficult for you to be involved in your child’s treatment this past week? (select all that apply) | □ Child does not want me to be involved  □ Therapist did not ask me to participate  □ I was not told how I could participate  □ Relationship problem with my child  □ Poor communication with my child  □ Child’s problems are too difficult for me to handle  □ I felt blamed by the therapist for my child’s problems  □ My work schedule  □ Transportation problems  □ Custody or placement situation  □ I have too many other things to do  □ My mental or physical health problems  □ I am too stressed  □ Therapy is not in my primary language  □ Other (please specify) |
| 1. This past week, I wanted to be involved in my child’s therapy. | (1) Strongly disagree to (5) strongly agree |
| 1. This past week, I wanted to learn new ways for managing my child’s behavior. | (1) Strongly disagree to (5) strongly agree |
| 1. This past week, I was motivated to work with my child’s therapist. | (1) Strongly disagree to (5) strongly agree |
| 1. This past week, I believed I could learn skills to change my child’s behavior. | (1) Strongly disagree to (5) strongly agree |
| 1. This past week, I believed my child’s behavior would change if I were involved in their therapy. | (1) Strongly disagree to (5) strongly agree |
| 1. This past week, I believed changing my behavior would change my child’s behavior. | (1) Strongly disagree to (5) strongly agree |
| **NOTE:** All participants will continue with Q12. | |
| 1. How long was your child’s therapy session? | (Hour and minute response option) |
| **NOTE**: Instructions for Q13-Q18. Please answer the following questions based on your relationship with your child’s therapist over the past week, even if you did not attend or participate in your child’s therapy session. | |
| 1. My child’s therapist and I worked towards goals we both agree on. | 0 (not at all) 1 (a little) 2 (moderately) 3 (quite a bit), 4 (very much), 5 (completely) |
| 1. My child’s therapist and I agreed on what is important for my child to work on. | 0 (not at all) 1 (a little) 2 (moderately) 3 (quite a bit), 4 (very much), 5 (completely) |
| 1. My child’s therapist and I respected each other. | 0 (not at all) 1 (a little) 2 (moderately) 3 (quite a bit), 4 (very much), 5 (completely) |
| 1. I believe my child’s therapist liked me. | 0 (not at all) 1 (a little) 2 (moderately) 3 (quite a bit), 4 (very much), 5 (completely) |
| 1. I believe the things I do in my child’s therapy will help to accomplish the changes that I want for my child. | 0 (not at all) 1 (a little) 2 (moderately) 3 (quite a bit), 4 (very much), 5 (completely) |
| 1. I felt that my child’s therapist would care about me if I did things they do not approve of. | 0 (not at all) 1 (a little) 2 (moderately) 3 (quite a bit), 4 (very much), 5 (completely) |
| **NOTE**: If the caregiver endorses being in the therapy session (“Yes” to Q3), they will answer Q19-Q24. All participants will continue with Q19. | |
| 1. How did YOUR CHILD’S THERAPIST BEHAVE toward you during their therapy session this past week? | -50 (Distant/Cold) to 50 (Warm/Friendly) |
| 1. How did YOUR CHILD’S THERAPIST BEHAVE toward you during their therapy session this past week? | -50 (Shy/Passive) to 50 (Confident/Pushy) |
| 1. How did YOU BEHAVE toward your child’s therapist during their therapy session this past week? | -50 (Distant/Cold) to 50 (Warm/Friendly) |
| 1. How did YOU BEHAVE toward your child’s therapist during their therapy session this past week? | -50 (Shy/Passive) to 50 (Confident/Pushy) |
| 1. How helpful was your child’s therapy this past week? | 0 (not at all helpful) – 10 (extremely helpful) |
| 1. How much did you participate in your child’s therapy this past week? | Not At All (1) – Extremely (10) |
| **NOTE**: All participants will continue with Q19. | |
| 1. How important was your child’s therapy to YOU this past week? | Not important (1) – Extremely Important (10) |
| 1. How important was your child’s therapy to THEM this past week? | Not important (1) – Extremely Important (10) |
| 1. How motivated were you to participate in your child’ therapy this past week? | Not Motivated (1) – Extremely Motivated (10) |
| **NOTE**: Q28 will only be administered if a participant endorses being in the therapy session (“Yes” to Q3). If they answered “No” to Q3, they will proceed to Q30. | |
| 1. What did you talk about in your child’s therapy this week? (select all that apply) | □ Emotions, like anger, anxiety, or depression  □ Thoughts, like unhelpful thinking patterns  □ Stressors or traumas  □ Substance use or cravings  □ Friends, peers, or romantic partner  □ Parent or Caregiver  □ Other family members  □ School  □ Work  □ Activities/Hobbies  □ None of the above  □ Other (please specify) |
| **NOTE**: Q29 will be administered when a participant selects other to Q28. | |
| 1. What else did you talk about in your child’s therapy this week? | Open response |
| **NOTE**: Instructions for Q30-31. The following questions ask about things you may have learned or practiced from your child’s therapy this past week even if you did not attend or participate in their therapy (e.g., practice worksheets from therapist, things you learned or practiced from prior sessions). | |
| 1. What did you learn or practice in your child’s therapy this week? (select all that apply) | □ why my child is in therapy  □ better ways for me and my child to talk  □ better ways for my child to talk with other family members  □ better ways for my child to talk with their friends or romantic partner  □ ways for my child to relax when having strong feelings  □ ways for my child to deal with their substance use  □ an activity to help my child face their fears like trauma, social anxiety, school anxiety  □ how to better solve problems  □ None of the above |
| 1. During your child’s therapy this week, did you learn or practice how… (select all that apply) | □ fun activities can improve my child’s mood  □ thoughts can change feelings and behaviors  □ to spot unhelpful thoughts or try more helpful ways of thinking  □ what happens before or after a behavior, like an argument or substance use, can help change my child’s future behavior  □ rewards and consequences can impact my child’s behavior  □ to do anything else not included in this list  □ None of the above |
| **NOTE**: If a participant selects “to do anything else not included in this list” they will answer Q32. | |
| 1. What did you learn or practice? | Open response |
| **NOTE**: For each answer selected in Q30 and Q31, participants will be asked Q33-35. | |
| 1. How helpful was learning or practicing [enter skill selected from above]? | 0 (not at all helpful) – 10 (extremely helpful) |
| 1. During your child’s therapy session, how much time did you spend learning or practicing [enter skill selected from above]? | (Hour and minute response option) |
| 1. [enter skill selected from above]: In which areas did you and your therapist talk about using this? (select all that apply) | □ Emotions, like anger, anxiety, or depression  □ Thoughts, like unhelpful thinking patterns  □ Stressors or traumas  □ Substance use or cravings  □ Friends, peers, or romantic partner  □ Parent or Caregiver  □ Other family members  □ School  □ Work  □ Activities/Hobbies  □ None of the above  □ Other (please specify) |
| **NOTE**: All participants will continue at Q36. | |
| 1. This past week, how much did you SUPPORT YOUR CHILD’S MENTAL HEALTH? | Not at all (0) to Very Much (10) |
| 1. This past week, how much did you SUPPORT YOUR CHILD’S SUBSTANCE USE GOALS? | Not at all (0) to Very Much (10) |
| 1. This past week, how much did you HELP YOUR CHILD PRACTICE WAYS OF MANAGING THEIR MENTAL HEALTH OR SUBSTANCE USE? | Not at all (0) to Very Much (10) |
| 1. This past week, how much did you ENCOURAGE YOUR CHILD TO ATTEND THERAPY? | Not at all (0) to Very Much (10) |
| 1. This past week, how much did your child ENGAGE IN FAMILY ACTIVITIES? | Not at all (0) to Very Much (10) |
| 1. This past week, how many days did you have family meals with your child. | 0 days – 7 days |
| 1. How much do you think therapy so far has helped your child with their SUBSTANCE USE? | 0 (not at all) to 10 (completely or extremely) |
| 1. How much do you think therapy so far has helped your child with their MENTAL HEALTH? | 0 (not at all) to 10 (completely or extremely) |
| 1. How much do you think therapy so far has helped your child RELATIONSHIP WITH YOU AND THEIR OTHER CAREGIVERS? | 0 (not at all) to 10 (completely or extremely) |
| 1. How much do you think therapy so far has helped your child’s RELATIONSHIPS WITH THEIR FRIENDS? | 0 (not at all) to 10 (completely or extremely) |
| 1. How much do you think therapy so far has helped your child with SCHOOL, EDUCATION, OR WORK ? | 0 (not at all) to 10 (completely or extremely) |
| 1. Check all the recovery supports you attended or used this past week? ) | □ Smart phone app – parenting practices  □ Smart phone app – mental health  □ Smart phone app – substance use  □ Parenting support group  □ Al-anon/Nar-anon meeting  □ Faith-based support  □ Other recovery support  □ I did not use another recovery support |
| **NOTE**: Participants will be asked Q48 if they select “Other recovery support” in Q47. For each recovery support selected, participants will be asked Q49-50. Participants who select “I did not use another recovery support” will continue with Q51. | |
| 1. What was the recovery support you used? | (open text) |
| 1. How many days did you use this support in the past week? | 1 days – 7 days |
| 1. How helpful was [pipe Q47 response]? | 0 (not at all helpful) – 10 (extremely helpful) |
| 1. This past week, did your child take any prescribed medication for their mental health or substance use? (select all that apply) | □ Yes, for their mental health  □ Yes, for their substance use  □ No  □ Not sure |
| **NOTE**: If a participant endorses “Yes” to Q51 they will answer Q52-54, if they answer “No” they will be directed to Question 55. | |
| 1. This past week, how many days did your child forget to take their medication? | 1 day - 7 days |
| 1. This past week, how much did you help your child with taking their medication? | Not at all (0) to Very Much (10) |
| 1. This past week, how helpful was this medication? | 0 (not at all helpful) – 10 (extremely helpful) |
| **NOTE**: All participants will continue with Q61. | |
| 1. Did you experience any of the following events during the past week. (select all that apply) | □ I was targeted or harassed  □ I saw or heard negative, hurtful, or offensive messages or stereotypes  □ I was ignored, isolated, or made to feel invisible  □ I was misunderstood  □ People stared at me  □ I was not accepted  □ Someone made me feel uncomfortable or unsafe  □ Someone made me feel less of a human  □ None of the above. |
| **NOTE**: Q56 will be administered for each response other than “None of the above” to Q51. | |
| 1. What do you think is the reason(s) for this experience? (select all that apply) | □ My sexual orientation  □ My gender identity  □ My gender expression  □ My race/ethnicity  □ My substance Use  □ My mental health  □ My body size  □ My physical Disability  □ My social class  □ Other (please specify) |

**Shortest Possible Length**: 29 items*

**Longest Possible Length**: 49 items*

**Mental Health Update Report Instructions, Timing Parameters, and General Functions**

1. The mental health update will be added to participant morning reports each Saturday.
2. Considering the mental health update will be added to morning reports, the same reminders for morning reports will be used for the mental health update reports.
3. If a participant does not complete the mental health update report on Saturday, it will be added to their morning report on Sunday.

# Mental Health Update Report – Caregiver (Did Not Have Therapy/Missed Session)

| **ITEM** | **RESPONSE OPTION** |
| --- | --- |
| **NOTE**: Q1 will be administered if a participant selects “No, they did not have therapy this week.” Q2 will be administered if a participant selects “No, they missed their appointment.” | |
| 1. What were the reason(s) for your child not having a therapy session this past week? (select all that apply) | □ They were too busy or had a conflict in their schedule  □ Their therapist was not available  □ They had transportation issues  □ They could not afford transportation  □ They could not afford the therapy session  □ They were physically sick  □ Their mental health symptoms  □ They did not want to talk to their therapist  □ They do not find therapy helpful  □ They had a conflict with me or another caregiver  □ They had family or personal problems  □ Other (please specify)  □ None of the above |
| 1. What were the reason(s) your child missed their therapy session this past week? (select all that apply) | □ They forgot about the session  □ I forgot about their session  □ They were too busy or had a conflict in their schedule  □ They had transportation issues  □ They could not afford transportation  □ They could not afford the therapy session  □ They were physically sick  □ Their mental health symptoms  □ They did not want to talk to their therapist  □ They do not find therapy helpful  □ They had a conflict with me or another caregiver  □ They had family or personal problems  □ Other (please specify)  □ None of the above |
| **Note**: Instructions for Q3-Q8. Please answer the following questions based on your relationship with your child’s therapist over the past week, even if they missed their session or did not have one. | |
| 1. My child’s therapist and I worked towards goals we both agree on. | 0 (not at all) 1 (a little) 2 (moderately) 3 (quite a bit), 4 (very much), 5 (completely) |
| 1. My child’s therapist and I agreed on what is important for my child to work on. | 0 (not at all) 1 (a little) 2 (moderately) 3 (quite a bit), 4 (very much), 5 (completely) |
| 1. My child’s therapist and I respected each other. | 0 (not at all) 1 (a little) 2 (moderately) 3 (quite a bit), 4 (very much), 5 (completely) |
| 1. I believe my child’s therapist liked me. | 0 (not at all) 1 (a little) 2 (moderately) 3 (quite a bit), 4 (very much), 5 (completely) |
| 1. I believe the things I do in my child’s therapy will help to accomplish the changes that I want for my child. | 0 (not at all) 1 (a little) 2 (moderately) 3 (quite a bit), 4 (very much), 5 (completely) |
| 1. I felt that my child’s therapist would care about me if I did things they do not approve of. | 0 (not at all) 1 (a little) 2 (moderately) 3 (quite a bit), 4 (very much), 5 (completely) |
| 1. Check all the recovery supports you attended or used this past week? ) | □ Smart phone app – parenting practices  □ Smart phone app – mental health  □ Smart phone app – substance use  □ Parenting support group  □ Al-anon/Nar-anon meeting  □ Faith-based support  □ Other recovery support  □ I did not use another recovery support |
| **NOTE**: Participants will be asked Q10 if they select “Other recovery support” in Q9. For each recovery support selected, participants will be asked Q11-12. Participants who select “I did not use another recovery support” will continue with Q13. | |
| 1. What was the recovery support you used? | (open text) |
| 1. How many days did you use this support in the past week? | 1 days – 7 days |
| 1. How helpful was this recovery support? | 0 (not at all helpful) – 10 (extremely helpful) |
| 1. This past week, did your child take any prescribed medication for their mental health or substance use? (select all that apply) | □ Yes, for their mental health  □ Yes, for their substance use  □ No  □ Not sure |
| **NOTE**: If a participant endorses “Yes” to Q13 they will answer Q14-16, if they answer “No” they will be directed to Q17. | |
| 1. This past week, how many days did your child forget to take their medication? | 1 day - 7 days |
| 1. This past week, how much did you help your child with taking their medication? | Not at all (0) to Very Much (10) |
| 1. This past week, how helpful was this medication? | 0 (not at all helpful) – 10 (extremely helpful) |
| **NOTE**: All participants continue with Q17. | |
| 1. How important was your child’s therapy to YOU this past week? | Not important (1) – Extremely Important (10) |
| 1. How important was your child’s therapy to THEM this past week? | Not important (1) – Extremely Important (10) |
| 1. How motivated were you to participate in your child’ therapy this past week? | Not Motivated (1) – Extremely Motivated (10) |
| 1. This past week, did you learn or practice… (select all that apply) | □ why my child is in therapy  □ better ways for me and my child to talk  □ better ways for my child to talk with other family members  □ better ways for my child to talk with their friends or romantic partner  □ ways for my child to relax when having strong feelings  □ ways for my child to deal with their substance use  □ an activity to help my child face their fears like trauma, social anxiety, school anxiety  □ how to better solve problems  □ None of the above |
| 1. This past week, did you learn or practice how… (select all that apply) | □ fun activities can improve my child’s mood  □ thoughts can change feelings and behaviors  □ to spot unhelpful thoughts or try more helpful ways of thinking  □ what happens before or after a behavior, like an argument or substance use, can help change my child’s future behavior  □ rewards and consequences can impact my child’s behavior  □ to do anything else not included in this list  □ None of the above |
| **NOTE**: If a participant selects “to do anything else not included in this list” they will answer Q22. | |
| 1. What did you learn or practice? | Open response |
| **NOTE**: For each answer selected in Q20 and Q21, participants will be asked Q23-25. | |
| 1. [enter skill selected from above]: How helpful was learning or practicing this? | 0 (not at all helpful) – 10 (extremely helpful) |
| 1. [enter skill selected from above]: During your child’s therapy session, how much time did you spend learning or practicing this? | (Hour and minute response option) |
| 1. [enter skill selected from above]: In which areas did you use this skill or help your child use this skill? (select all that apply) | □ Emotions (e.g., anger, anxiety, depression)  □ Thoughts (e.g., unhelpful thoughts)  □ Stressors or traumas  □ Substance use or cravings  □ Friends, peers, or romantic partner  □ Family  □ School  □ Work  □ Activities/Hobbies  □ Other |
| **NOTE**: All participants will continue at Q26. | |
| 1. This past week, how much did you SUPPORT YOUR CHILD’S MENTAL HEALTH? | Not at all (0) to Very Much (10) |
| 1. This past week, how much did you SUPPORT YOUR CHILD’S SUBSTANCE USE GOALS? | Not at all (0) to Very Much (10) |
| 1. This past week, how much did you HELP YOUR CHILD PRACTICE WAYS OF MANAGING THEIR MENTAL HEALTH OR SUBSTANCE USE? | Not at all (0) to Very Much (10) |
| 1. This past week, how much did you ENCOURAGE YOUR CHILD TO ATTEND THERAPY? | Not at all (0) to Very Much (10) |
| 1. This past week, how much did your child ENGAGE IN FAMILY ACTIVITIES? | Not at all (0) to Very Much (10) |
| 1. This past week, how many days did you have family meals with your child? | 0 days – 7 days |
| 1. How much do you think therapy so far has helped your child with their SUBSTANCE USE? | 0 (not at all) to 10 (completely or extremely) |
| 1. How much do you think therapy so far has helped your child with their MENTAL HEALTH? | 0 (not at all) to 10 (completely or extremely) |
| 1. How much do you think therapy so far has helped your child RELATIONSHIP WITH YOU AND THEIR OTHER CAREGIVERS? | 0 (not at all) to 10 (completely or extremely) |
| 1. How much do you think therapy so far has helped your child’s RELATIONSHIPS WITH THEIR FRIENDS? | 0 (not at all) to 10 (completely or extremely) |
| 1. How much do you think therapy so far has helped your child with SCHOOL, EDUCATION, OR WORK ? | 0 (not at all) to 10 (completely or extremely) |
| 1. Did you experience any of the following events during the past week. (select all that apply) | □ I was targeted or harassed  □ I saw or heard negative, hurtful, or offensive messages or stereotypes  □ I was ignored, isolated, or made to feel invisible  □ I was misunderstood  □ People stared at me  □ I was not accepted  □ Someone made me feel uncomfortable or unsafe  □ Someone made me feel less of a human  □ None of the above. |
| **NOTE**: Q38 will be administered for each response other than “None of the above” to Q37. | |
| 1. What do you think is the reason(s) for this experience? (select all that apply) | □ My sexual orientation  □ My gender identity  □ My gender expression  □ My race/ethnicity  □ My substance Use  □ My mental health  □ My body size  □ My physical Disability  □ My social class  □ Other (please specify) |

**Shortest Possible Length**: 26 items*

**Longest Possible Length**: 38 items*

*All participants will be administered Q1 from the mental health update report therapy session.

**Mental Health Update Report Instructions, Timing Parameters, and General Functions**

1. Participants are instructed to complete mental health update (MHU) after each therapy session.
2. The MHU is available at all times.
3. For participants who miss a therapy session or are no longer in treatment, they will be asked to complete MHU by Friday each week.
4. If the participant does not complete the MHU by Friday at 4pm, then a push notification will remind the participant to complete the MHU. Otherwise, the MHU will be embedded into the Wake-Up Report on Saturday.
5. This survey will be available the day the participant enrolls in project MAPP.

# Mental Health Update Report – Caregiver (quit or finished therapy)

| **ITEM** | **RESPONSE OPTION** |
| --- | --- |
| **NOTE**: Participants who select “No, I quit therapy early” will answer Q1 and Q2. Participants who select “No, I finished therapy” will continue with Q3. | |
| 1. Why is your child not currently in treatment? (select all that apply) | □ Their problem(s) went away  □ They wanted to handle their problem on their own  □ They thought the problem would get better  □ They did not know who to see  □ They were scared of what others would think of them being in treatment  □ They thought treatment would not work  □ They thought treatment would take too much time  □ Their problem(s) did not bother them much  □ They were scared on being involuntarily hospitalized  □ Their past experiences in therapy were not helpful  □ They were unhappy with the treatment they were receiving  □ None of the above |
| 1. Is your child not currently in treatment for any of the following reasons? (select all that apply) | □ I did not support my child going to therapy  □ Issues in our family prevented my child from going to therapy  □ They could not get an appointment  □ Treatment was too expensive  □ Transportation to treatment was too expensive  □ Treatment was not covered by insurance  □ They had a hard time getting transportation to treatment  □ It was too hard to find a time for treatment each week  □ None of the above |
| 1. Check all the recovery supports you attended or used this past week? ) | □ Smart phone app – parenting practices  □ Smart phone app – mental health  □ Smart phone app – substance use  □ Parenting support group  □ Al-anon/Nar-anon meeting  □ Faith-based support  □ Other recovery support  □ I did not use another recovery support |
| **NOTE**: Participants will complete Q4 if they endorse “other recovery support” in Q3. Participants will complete Q5 and Q6 for each response in Q3 other than “I did not use another recovery support.” Participants will skip to Q7 if they endorse “I did not use another recovery support.” | |
| 1. What was the recovery support you used? | (open text) |
| 1. How many days did you use this support in the past week? | 1 days – 7 days |
| 1. How helpful was this recovery support? | 0 (not at all helpful) – 10 (extremely helpful) |
| 1. This past week, did your child take any prescribed medication for their mental health or substance use? (select all that apply) | □ Yes, for their mental health  □ Yes, for their substance use  □ No  □ Not sure |
| **NOTE**: If a participant endorses “Yes” to Q7 they will answer Q8-10, if they answer “No” they will be directed to Q11. | |
| 1. This past week, how many days did your child forget to take their medication? | 1 day - 7 days |
| 1. This past week, how much did you help your child with taking their medication? | Not at all (0) to Very Much (10) |
| 1. This past week, how helpful was this medication? | 0 (not at all helpful) – 10 (extremely helpful) |
| 1. This past week, did you learn or… (select all that apply) | □ better ways for me and my child to talk  □ better ways for my child to talk with other family members  □ better ways for my child to talk with their friends or romantic partner  □ ways for my child to relax when having strong feelings  □ ways for my child to deal with their substance use  □ an activity to help my child face their fears like trauma, social anxiety, school anxiety  □ how to better solve problems  □ None of the above |
| 1. This past week, did you learn or practice how… (select all that apply) | □ fun activities can improve my child’s mood  □ thoughts can change feelings and behaviors  □ to spot unhelpful thoughts or try more helpful ways of thinking  □ what happens before or after a behavior, like an argument or substance use, can help change my child’s future behavior  □ rewards and consequences can impact my child’s behavior  □ to do anything else not included in this list  □ None of the above |
| **NOTE**: If a participant selects “to do anything else not included in this list” they will answer Q13. | |
| 1. What did you learn or practice? | Open response |
| **NOTE**: For each answer selected in Q11-Q12, participants will be asked Q14-16. If they select “None of the above” for Q11 and Q12 they will continue with Q17. | |
| 1. [enter skill selected from above]: How helpful was learning or practicing this? | 0 (not at all helpful) – 10 (extremely helpful) |
| 1. [enter skill selected from above]: During your child’s therapy session, how much time did you spend learning or practicing this? | (Hour and minute response option) |
| 1. [enter skill selected from above]: In which areas did you use this skill or help your child use this skill? (select all that apply) | □ Emotions (e.g., anger, anxiety, depression)  □ Thoughts (e.g., unhelpful thoughts)  □ Stressors or traumas  □ Substance use or cravings  □ Friends, peers, or romantic partner  □ Family  □ School  □ Work  □ Activities/Hobbies  □ Other |
| **NOTE**: All participants will continue with Q17. | |
| 1. This past week, how much did you SUPPORT YOUR CHILD’S MENTAL HEALTH? | Not at all (0) to Very Much (10) |
| 1. This past week, how much did you SUPPORT YOUR CHILD’S SUBSTANCE USE GOALS? | Not at all (0) to Very Much (10) |
| 1. This past week, how much did you HELP YOUR CHILD PRACTICE WAYS OF MANAGING THEIR MENTAL HEALTH OR SUBSTANCE USE? | Not at all (0) to Very Much (10) |
| 1. This past week, how much did you ENCOURAGE YOUR CHILD TO ATTEND THERAPY? | Not at all (0) to Very Much (10) |
| 1. This past week, how much did your child ENGAGE IN FAMILY ACTIVITIES? | Not at all (0) to Very Much (10) |
| 1. This past week, how many days did you have family meals with your child? | 0 days – 7 days |
| 1. Did you experience any of the following events during the past week. (select all that apply) | □ I was targeted or harassed  □ I saw or heard negative, hurtful, or offensive messages or stereotypes  □ I was ignored, isolated, or made to feel invisible  □ I was misunderstood  □ People stared at me  □ I was not accepted  □ Someone made me feel uncomfortable or unsafe  □ Someone made me feel less of a human  □ None of the above. |
| **NOTE**: Q24 will be administered for each response other than “None of the above” to Q25. | |
| 1. What do you think is the reason(s) for this experience? (select all that apply) | □ My sexual orientation  □ My gender identity  □ My gender expression  □ My race/ethnicity  □ My substance Use  □ My mental health  □ My body size  □ My physical Disability  □ My social class  □ Other (please specify)  □ None of the above |

**Shortest Possible Length**: 13 items*

**Longest Possible Length**: 25 items*

*All participants will be administered Q1 from the mental health update report therapy session.

**Mental Health Update Report Instructions, Timing Parameters, and General Functions**

1. The mental health update will be added to participant morning reports each Saturday.
2. Considering the mental health update will be added to morning reports, the same reminders for morning reports will be used for the mental health update reports.
3. If a participant does not complete the mental health update report on Saturday, it will be added to their morning report on Sunday.

# Mental Health Update Report – Caregiver (not sure if child had a therapy session)

| **ITEM** | **RESPONSE OPTION** |
| --- | --- |
| 1. Why are you not sure if you child had therapy this past week: (select all that apply) | □ My child and I do not speak about their therapy  □ My child and I are not speaking to each other right now  □ My child was not at home much this week  □ My child is living with someone else right now  □ I was out of town this week  □ My work hours  □ A reason not listed above |
| **NOTE**: Q2 will be administered if a participant endorses “A reason not listed above” to Q1. | |
| 1. What was the reason? | Open response |
| 1. Check all the recovery supports you attended or used this past week? | □ Smart phone app – parenting practices  □ Smart phone app – mental health  □ Smart phone app – substance use  □ Parenting support group  □ Al-anon/Nar-anon meeting  □ Faith-based support  □ Other recovery support  □ I did not use another recovery support |
| **NOTE**: Participants will be asked Q4 if they select “Other recovery support” in Q3. For each recovery support selected, participants will be asked Q5 and Q6. Participants who select “I did not use another recovery support” will continue with Q7. | |
| 1. What was the recovery support you used? | (open text) |
| 1. How many days did you use this support in the past week? | 1 days – 7 days |
| 1. How helpful was this recovery support? | 0 (not at all helpful) – 10 (extremely helpful) |
| 1. This past week, did your child take any prescribed medication for their mental health or substance use? (select all that apply) | □ Yes, for their mental health  □ Yes, for their substance use  □ No  □ Not sure |
| **NOTE**: If a participant endorses “Yes” to Q7 they will answer Q8-10, if they answer “No” they will be directed to Q11. | |
| 1. This past week, how many days did your child forget to take their medication? | 1 day - 7 days |
| 1. This past week, how much did you help your child with taking their medication? | Not at all (0) to Very Much (10) |
| 1. This past week, how helpful was this medication? | 0 (not at all helpful) – 10 (extremely helpful) |
| **NOTE**: All participants will continue with Q11. | |
| 1. This past week, did you learn or… (select all that apply) | □ better ways for me and my child to talk  □ better ways for my child to talk with their other family members, friends, or others  □ ways for my child to relax when having strong feelings  □ ways for my child to deal with their substance use  □ an activity to help my child face their fears like trauma, social anxiety, school anxiety  □ how to better solve problems  □ None of the above |
| 1. This past week, did you learn or practice how… (select all that apply) | □ fun activities can improve my child’s mood  □ thoughts can change feelings and behaviors  □ to spot unhelpful thoughts or try more helpful ways of thinking  □ what happens before or after a behavior, like an argument or substance use, can help change my child’s future behavior  □ rewards and consequences can impact my child’s behavior  □ to do anything else not included in this list  □ None of the above |
| **NOTE**: If a participant selects “to do anything else not included in this list” they will answer Q13. | |
| 1. What did you learn or practice? | Open response |
| **NOTE**: For each answer selected in Q11 and 12, participants will be asked Question Q14-Q16. | |
| 1. [enter skill selected from above]: How helpful was learning or practicing this? | 0 (not at all helpful) – 10 (extremely helpful) |
| 1. [enter skill selected from above]: During your child’s therapy session, how much time did you spend learning or practicing this? | (Hour and minute response option) |
| 1. [enter skill selected from above]: In which areas did you use this skill or help your child use this skill? (select all that apply) | □ Emotions (e.g., anger, anxiety, depression)  □ Thoughts (e.g., unhelpful thoughts)  □ Stressors or traumas  □ Substance use or cravings  □ Friends, peers, or romantic partner  □ Family  □ School  □ Work  □ Activities/Hobbies  □ Other |
| **NOTE**: All participants will continue with Q17. | |
| 1. This past week, how much did you SUPPORT YOUR CHILD’S MENTAL HEALTH? | Not at all (0) to Very Much (10) |
| 1. This past week, how much did you SUPPORT YOUR CHILD’S SUBSTANCE USE GOALS? | Not at all (0) to Very Much (10) |
| 1. This past week, how much did you HELP YOUR CHILD PRACTICE WAYS OF MANAGING THEIR MENTAL HEALTH OR SUBSTANCE USE? | Not at all (0) to Very Much (10) |
| 1. This past week, how much did you ENCOURAGE YOUR CHILD TO ATTEND THERAPY? | Not at all (0) to Very Much (10) |
| 1. This past week, how much did your child ENGAGE IN FAMILY ACTIVITIES? | Not at all (0) to Very Much (10) |
| 1. This past week, how many days did you have family meals with your child? | 0 days – 7 days |
| 1. Did you experience any of the following events during the past week. (select all that apply) | □ I was targeted or harassed  □ I saw or heard negative, hurtful, or offensive messages or stereotypes  □ I was ignored, isolated, or made to feel invisible  □ I was misunderstood  □ People stared at me  □ I was not accepted  □ Someone made me feel uncomfortable or unsafe  □ Someone made me feel less of a human  □ None of the above. |
| **NOTE:** Q24 will be administered for each response other than “None of the above” to Q23. | |
| 1. What do you think is the reason(s) for this experience? (select all that apply) | □ My sexual orientation  □ My gender identity  □ My gender expression  □ My race/ethnicity  □ My substance Use  □ My mental health  □ My body size  □ My physical Disability  □ My social class  □ Other (please specify)  □ None of the above |

**Shortest Possible Length**: 13 items*

**Longest Possible Length**: 25 items*

*All participants will be administered Q1 from the mental health update report therapy session.
