# Supplementary material for: Examining Caregiver Practices During Adolescent Outpatient Alcohol Use and Co-Occurring Mental Health Treatment: Protocol for a Dyadic Ecological Momentary Assessment Study
Source: JMIR Res Protoc. 2024 Dec 20;13:e63399. doi: 10.2196/63399 (PMC11702013; doi:10.2196/63399)
Supplement: Multimedia Appendix 2 [file resprot_v13i1e63399_app2.docx]

|  |  |
| --- | --- |
|  | **D** |

**Figure 1**. K99 Pilot Study Ecological Momentary Assessment Accountable Compliance for Adolescents and Caregivers
